# Metal Aerogel Electrocatalysts for Methanol Oxidation Reaction in Direct Methanol Fuel Cells: A Comprehensive Review on Progress, Performance, and Future Perspectives

**DOI:** 10.3390/gels12070575

**Published:** 2026-06-29

**Authors:** Shaik Ashmath, Mohanraj Vinothkannan, Bhim Sen Thapa, Myunghwan Byun, Shaik Gouse Peera

**Affiliations:** 1 Department of Environmental Engineering, Keimyung University, 1095 Dalgubeol-daero, Dalseo-gu, Daegu 42601, Republic of Korea; shaikashmath2@gmail.com; 2 Department of Chemistry, Karpagam Academy of Higher Education, Deemed University, Coimbatore 641021, Tamil Nadu, India; 3 Centre for Advanced Low Carbon Propulsion Systems (C-ALPS), Centre for E-Mobility and Clean Growth, Coventry University, Coventry CV1 2TL, UK; ae4313@coventry.ac.uk; 4 Department of Biological Sciences, Marquette University, Milwaukee, WI 53233, USA; bhimsen.thapa@marquette.edu; 5 Department of Advanced Materials Engineering, Keimyung University, 1095 Dalgubeol-daero, Dalseo-gu, Daegu 42601, Republic of Korea; myunghbyun@kmu.ac.kr

**Keywords:** self-supported electrocatalysts, methanol oxidation, DMFCs, metal aerogels, mass activity, sol-gel, gelation

## Abstract

Direct methanol fuel cells (DMFCs) have attracted considerable attention recently for various applications ranging from portable ones to transportation. The efficiency of DMFCs depends on the kinetics of anodic and cathodic electrocatalysts. Due to sluggish anodic methanol oxidation reaction (MOR), DMFCs require an effective and bifunctional catalyst for promoting efficient MOR. The state-of-the-art MOR catalysts, such as Pt/C and Pt-Ru/C, have been shown to exhibit reasonable MOR activity; however, the insufficient mass activity and poor stability of carbon-supported catalysts have been a major limitation, requiring an alternative, efficient, electrocatalyst that exhibits high mass and specific activities. In addition, electrocatalysts without any carbon support (self-supported electrocatalysts) further mitigate their poor stability and therefore enhance their durability. In this regard, metal aerogel catalysts, which are entirely composed of metallic networks, recently attained special interest due to their specific advantages over conventional carbon supports, such as high catalyst utilization and improved electronic conductivity and stability. In this review, we systematically reviewed various metal aerogel catalysts developed for MOR since their first discovery in 2009. The metal aerogel demonstrated superior MOR performance relative to carbon-supported commercial catalysts, with enhancements ranging from 2-fold to 22-fold of mass activity. We also statistically compared the mass activity of metal aerogels with traditional carbon-supported, non-carbon-supported, and advanced shape-controlled catalysts and found that metal aerogels exhibited high mass activities compared to other catalyst systems. Therefore, we clearly establish that metal aerogel catalysts possess great potential as efficient MOR catalysts in DMFCs. In addition, we have provided several future research directions and strategies for further development of metal aerogel-integrated DMFC devices.

## 1. Introduction

With increasing global population growth and energy demand, high-performance energy-producing devices such as electric vehicles, batteries, supercapacitors, and power grids, as well as portable devices such as laptops, smartphones, etc., have recently become increasingly demanding. On the other hand, conventional fossil fuel-based energy sources not only harm the environment by releasing various greenhouse gases but also are non-renewable. Therefore, energy devices that work on renewable fuel sources have gained tremendous interest in recent times. In this regard, liquid-based direct methanol fuel cells (DMFCs) have attracted considerable interest due to their multiple advantages, such as the renewable nature of methanol fuel, low fuel cost, high energy density, fuel safety, and easier storage and transport of the fuel [1,2]. DMFCs are interesting in terms of their fuel availability, production, storage, and safe transport when compared to highly flammable, hydrogen-based fuel cells, which require high safety standards [3]. In addition, the methanol fuel has a higher volumetric energy density than gaseous hydrogen (4820 Wh/L vs. 180 Wh/L). DMFCs also possess several advantages over conventional lithium-based batteries, such as a higher gravimetric energy density (600 Wh/kg vs. 200 Wh/kg), easier refueling, and the ability to operate in an open system with a continuous fuel supply [4]. Therefore, DMFC-based fuel cells for energy conversion could be convenient for various portable, stationary, and mobile applications.

### 1.1. Operating Principle of DMFCs

**Figure 1:**
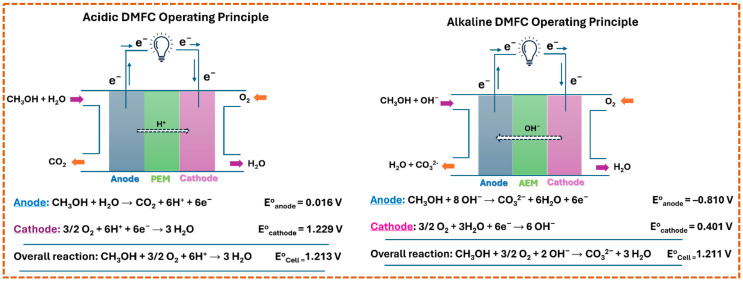
Operating principle of DMFC in acidic and alkaline electrolytes.

A typical DMFC operates with liquid methanol as anodic fuel and atmospheric air (O_2_) or pure oxygen as cathodic oxidant. The DMFCs can operate with either acidic pH environments (aq. methanol + aq. H_2_SO_4_) or in alkaline pH environments (aq. methanol + aq. NaOH/KOH). In either case, the liquid methanol fuel undergoes oxidation at the anode, and O_2_ undergoes reduction at the cathode. In an acidic pH environment, on the catalyst surface, methanol undergoes oxidation to form H^+^, e^−^, and CO_2_. The e^−^ moves from anode to cathode via the external circuit, the H^+^ passes through from anode to cathode via the proton exchange membrane, and the CO_2_ passes through the porous carbon and is released into the environment via the anode vent. On the cathode side, the O_2_ combines with the H^+^ and e^−^ to form H_2_O. In an alkaline pH environment, the methanol oxidizes to CO_2_ and electrons, while the cathodic O_2_ receives the electrons and converts them into OH^−^ in the presence of H_2_O [5]. The schematic representation of DMFCs in acidic and alkaline electrolytes is given in Figure 1 and Table 1.

**Figure 2:**
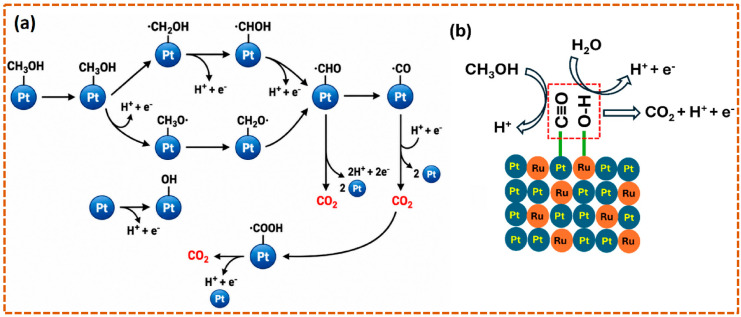
(**a**) Mechanism of methanol oxidation on the Pt surface [obtained from Ref. [5] (open access)], (**b**) Bifunctional mechanism of -CO oxidation on the Pt surface assisted by Ru.

As evidence of their viability and technological maturity, DMFCs have been used in a wide range of devices to date, including portable electronics, hearing aids, smoke detectors, sensors, automotive, and more [6,7,8,9]. Despite this, a few significant obstacles still stand in the way of the broader commercialization of DMFCs, especially for high-power demanding applications: (i) the methanol crossover problem; (ii) the slow anode reaction kinetics; and (iii) issues with heat, water, oxygen, and carbon dioxide management. Following is the sequence of electrochemical reactions that occur on the Pt catalyst surface (Figure 2a) and the electrochemical reactions, as shown below.

Mechanism of MOR in acidic electrolytes on the Pt surface

CH_3_OH + Pt → Pt–CH_2_OH + H^+^ + e^−^

Pt–CH_2_OH + Pt → Pt_2_–CHOH + H^+^ + e^−^

Pt_2_–CHOH + Pt → Pt_3_–CHO+ H^+^ + e^−^

Pt_3_–CHO → Pt_2_–CO + Pt + H^+^ + e^−^

Pt_2_–CO + H_2_O → Pt–COOH + Pt + H^+^ + e^−^

Pt–COOH → Pt + CO_2_ + H^+^ + e^−^

Mechanism of MOR in alkaline electrolytes on the Pt surface

Pt + OH^−^ → Pt–OH + e^−^

Pt–CH_3_OH + OH^−^ → Pt–CH_3_O + H_2_O + e^−^

Pt–CH_3_O + OH^−^ → Pt–CH_2_O + H_2_O + e^−^

Pt–CH_2_O + OH^−^ → Pt–CHO + H_2_O + e^−^

Pt–CHO + Pt–OH + 2OH^−^ → 2Pt + CO_2_ + 2H_2_O + e^−^

Bifunctional –CO species removal on the Pt-Ru catalytic system

Ru + H_2_O → Ru–OH + H^+^ + e^−^

Pt–CO + Ru–OH → Pt + Ru + CO_2_ + H^+^ + e^−^

The Pt/C catalyst’s strong absorption of CO intermediates, which prevents the Pt surface from undergoing additional methanol oxidation, is one of its greatest disadvantages for MOR. It is generally accepted that alloying the Pt with the metals that fulfill the requirement of forming O-containing surface species (–OH) at low potentials is a widely acceptable solution for CO removal from the Pt surface. Among them, Re, Sn, Ge, Mo, and Ru have been extensively investigated [10]. However, several of these metals exhibited destructive effects on methanol adsorption or suffered from low stability. Among these metals, Ru has been generally considered to have a balancing effect on –CO removal from the Pt surface and considerable stability with Pt [11]. Therefore, to date, Pt-Ru alloy electrocatalyst has been considered as a promising alternative to Pt/C on DMFC anodes. A popular bifunctional mechanism has been proposed for a synergistic mechanism of adsorbed CO removal from the Pt surface assisted by Ru atoms, as shown below (Figure 2b). This proposed bifunctional mechanism is based on the observation that at potentials below 0.4 V, Pt serves as an effective catalyst for methanol adsorption, yet is ineffective for water dissociation, whereas Ru can dissociate water but is incapable of adsorbing methanol. To date, Pt-Ru catalyst serves as a state-of-the-art catalyst for DMFC anodes. Very recently, it was proposed that a combination of bifunctional and electronic effects of Pt-Ru catalyst systems affects the efficient -CO removal [12].

### 1.2. Performance Metrics for DMFC

(a) *Half-Cell Performance Metrics*

The DMFCs performance analysis in a single cell is quite a tedious and time-consuming process. Furthermore, the DMFCs testing in a single cell requires a lot of materials such as carbon papers, membranes, hot-pressing high current load instruments, all of them associated with high cost. Furthermore, there are several engineering factors in membrane electrode assemblies (MEAs) that often complicate the testing process. Therefore, several researchers chose an alternate, more efficient, and cost-effective method of evaluating the MOR activity of the developed catalyst via traditional three-electrode-half-cell analysis. This consists of a glassy carbon electrode (GCE) that serves as a working electrode and substrate for catalyst deposition, a reference electrode, and a counter electrode. The catalyst powders are generally dispersed in water or a water-ethanol/isopropyl alcohol mixture and ultrasonicated to form the ink. A part of the ink is deposited on the GC electrode by a drop-casting method to make the working electrode. The MOR kinetics are evaluated in a mixture of methanol in acidic electrolyte or basic electrolyte, and a series of tests and performance metrics are followed as described below.

1. *Electrochemical Surface Area (ECSA) measurements:* ECSA is the total area of the electrode that is electrochemically active towards the MOR, which is usually measured by recording cyclic voltammetry curves (CV). For Pt and Rh-based catalysts, ECSA values (m^2^ g^−1^) are generally determined by integrating the region of hydrogen underpotential deposition (HUPD) and dividing by a constant of 210 μC cm^−2^ for Pt and 220 μC cm^−2^ (Rh) for the hydrogen monolayer and the mass of noble metal loaded on the glassy carbon electrode [13,14]. In addition to the traditional CV methods, the carbon monoxide (CO) stripping analysis is also a popular method to measure the ECSA of the noble metal catalyst. In the CO-stripping method, a charge associated with the CO oxidation will be used for Pt-based catalysts, which is 420 μC cm^−2^, and for Rh-based catalysts, it is 440 μC cm^−2^ [15]. For Pd-based catalysts, the area under the oxide-desorption peak is considered rather than the HUPD, and a monolayer oxide-adsorption charge value of 405 μC cm^−2^ is used for ECSA measurements, which is normalized by the Pd catalyst loading [16]. The ECSA measurements serve a very basic and fundamental way of understanding the active surface area of the catalysts that helps in optimizing the synthesis parameters and catalyst composition tuning for enhanced MOR performance.
2. *MOR performance analysis by I_F_ to I_R_ ratio:* The performance of the developed catalysts is evaluated relative to the baseline/standard catalyst by measuring the I_F_ to I_R_ ratio. MOR performance is usually assessed by CV, which results in two prominent peaks, one appearing during the forward scan and the other during the backward scan. The forward peak potentials represent the potentials at which the maximum methanol oxidation happens, and at that potential, a high anodic peak current is observed (*I_F_*). The CO that is formed during the forward MOR scan oxidizes during the reverse scan (*I_R_*). Many researchers believe that the peak current ratio of forward and backward/reverse scan (I_F_/I_R_) is taken as a tool to measure the catalyst potential [17]. Generally, a higher I_f_/I_R_ ratio indicates better catalytic activity, CO-tolerance, and oxophilicity of the electrocatalyst [18].
3. *Specific activity (mA cm^−2^) and mass activity (A mg^−1^):* The specific activity is defined as methanol oxidation current normalized by the ECSA (forward oxidation peak current, *I_F_*). The specific activity is obtained by dividing the methanol oxidation current by the ECSA. Mass activity is defined as the methanol oxidation current normalized by the catalyst loading (forward oxidation peak current, *I_F_*). Both specific and mass activities are important metrics for methanol oxidation electrocatalysis that indicate intrinsic activity, kinetics, and catalyst utilization, respectively. The specific activity of the electrocatalyst depends on the electronic structure, surface chemistry, composition of the catalyst, ligand, and lattice strain of the alloy catalysts. On the other hand, mass activity depends on the dispersion of the catalytically active sites and particle size of the electrocatalyst [19].
4. *MOR onset potential:* The onset potential is defined as the potential at which the oxidation current starts to appear for the forward CV scan. Although there is no universal definition for onset potential, it is commonly accepted that “onset potential is the potential at 5% of the MOR *I_F_* peak current”. The other comparative definition includes “the potential to reach a certain mass normalized current of 0.1 A mg^−1^, or geometric current density of 0.1 mA cm^−2^”.
5. *Stability:* The stability of the electrocatalyst is a very important aspect for long-term operation and the commercial applications of DMFCs. Generally, two types of stability tests are widely seen in the literature: (i) potential cycling test and (ii) chronoamperometric test. The potential cycling test normally employs repeated CV for several cycles (for instance, 1000–10,000 cycles) either in acidic/alkaline electrolytes with or without methanol. The normal potential window is from 0 V to 1.23 V vs. RHE for investigating the stability of the supported catalysts against metal dissolution, re-deposition, agglomeration, and coalescence, whereas the higher potential window up to 1.5 V is used for investigating the effect of support materials. The stability of the catalyst is assessed by the difference in the peak potentials, onset potentials before and after the stability test. Another popular stability test is the chronoamperometric (CA) test. It is a stability test in which the working electrode is held at a fixed potential (usually between 0.5–0.7 V vs. RHE) for long periods of time (usually hours to days) to measure the current response. Over time, when the current response remains constant, that indicates high stability of the catalysts.

(b) *The Membrane Electrode Assembly (MEA): The core of single-cell DMFCs*

The MEA is the heart of DMFCs. The MEA consists of an anode, a cathode, and the membrane. The anode consists of a solid substrate, gas diffusion layer, and the anode catalysts, and similarly, the cathode consists of a solid substrate, gas diffusion layer, and the cathode catalyst. The most common anode and cathode substrate in MEA is carbon paper. Carbon paper is coated with a gas diffusion layer (GDL) that comprises a carbon + PTFE mixture to maintain the balance of hydrophilic and hydrophobic nature of the carbon paper and helps to diffuse the liquid methanol (removal of CO_2_) and gaseous reactants at the cathodes, respectively. On the anode GDL, an anode catalyst is deposited or coated that acts as a reaction layer. The most common anode catalyst in DMFCs is Pt-Ru/C. The anode catalyst also contains the ionomer (Nafion in acidic DMFC and alkaline ionomer in alkaline DMFC), which acts as a binder that creates an effective three-phase contact in the catalyst layer to maximize the catalytic active sites utilization. Similarly, at the cathode side, the Pt/C catalyst is deposited, which serves as a reaction layer at the cathode that reduces the O_2_ to H_2_O. The MEAs are made by keeping the polymeric electrolyte membrane (ex. Nafion 115) in the center with the anode and cathodes positioned on opposite sides to one another, facing the catalyst layers towards the membrane, similar to a sandwiched structure, and are usually hot-pressed to make the final MEAs. The MEAs are then placed on the graphite flow fields to assemble into single-cell DMFCs. The single-cell DMFCs are supplied with aqueous methanol (in acidic/alkaline electrolytes) at the anode side via a peristaltic pump, and at the cathode side, the O_2_ (atmospheric air) is supplied. During the electrochemical reaction, the methanol is oxidized on the anode catalyst layer into H^+^ and e^−^ and the H^+^ ions pass through the Nafion membrane from anode to cathode. At the cathode H^+^ ions, electrons that pass through the external circuit from anode to cathode combine with the O_2_ to form H_2_O. The single-cell DMFCs are then analyzed for various electrochemical properties such as

1. *Polarization curves (I vs. V):* One of the primary objectives of fabricating the MEAs is to measure the cell voltage vs. current density of DMFCs to measure the maximum power density one can obtain from the anode catalysts [20]. In general, for comparative purposes, Pt/C is used as a cathode catalyst and Pt-Ru/C as an anode catalyst. The performance in terms of maximum power density (mW cm^−2^) is derived from the obtained voltage vs. current density values, and the relative efficiency of the newly developed anode catalyst is compared with the performance obtained with the Pt-Ru/C catalyst.
2. *Electrochemical Impedance Spectroscopy (EIS):* The EIS is generally employed to measure various resistances in DMFCs, such as Ohmic resistance (originated from the membrane and the interfacial contact between the electrode and membrane), charge transfer resistance (R_ct_, originates from kinetic resistance at anodes and cathodes) and mass transfer resistance (originates from the rate of mass transfer of reactants and removal of products, cathode flooding), and methanol crossover analysis [21].
3. *Performance mapping analysis:* The maximum power density of DMFCs with MEAs can be obtained by analyzing the single-cell performance at different temperatures (RT to 80 °C), different methanol concentrations (from 0.5–5.0 M), different flow rates of methanol to the anodes, and different flow rates of O_2_ (air) to the cathodes.
4. *Potentiostatic/Galvanostatic load cycling tests:* The MEAs in DMFCs can be analyzed to test their suitability in real-world applications by measuring the stability of the catalysts by holding at a fixed potential/current, and losses in the potential/current are measured over a fixed period of time (several hours to days and even longer) [22].
5. *Cyclic voltammetry (CV) for measuring ECSA:* CV is a fantastic tool to measure the ECSA of the catalyst, both at anodes and cathodes, by passing the inert gas at the measuring electrode and H_2_ gas at the opposite electrode. This method provides information on the ECAS to optimize the catalyst synthesis, loading catalyst coating methods, etc. [23].
6. *Methanol crossover measurements:* The methanol crossover is a serious concern that limits the overall efficiency of the DMFCs due to mixed potentials that occur at the cathodes. It is usually measured by performing linear sweep voltammetry at the cathodes with a methanol feed at the anode. If higher cathodic currents are seen in LSV, this indicates a higher methanol crossover. This is usually performed to analyze the effectiveness of the membrane [24].

## 2. Anode Electrocatalysts for MOR

By far, Pt-based catalysts (Pt supported on high surface area carbon, Pt/C) are considered as the state-of-the-art catalysts for MOR due to their suitable adsorption energy of methanol and easier oxidation capability [25]. The electrocatalytic activity of Pt-based catalysts depends on the morphology, composition, crystallinity, and facets. Several Pt-based catalysts have been investigated, such as Pt nanoparticles, nano frames, nano cubes, nanowires, nano sponges, and nanoflowers [26]. Despite this, Pt-based catalysts still suffer from drawbacks such as sluggish MOR kinetics, high cost, scarcity, and CO poisoning. Among these, CO poisoning is considered one of the serious problems that results in drastically reducing the electrocatalytic activity, which essentially requires a secondary metal to assist in eliminating the adsorbed CO from the Pt surface [27]. The secondary metal not only weakens the bond between Pt and CO but also facilitates the formation of oxygenated species such as OH_ads_ from the H_2_O decomposition that helps to oxidize the adsorbed CO species into CO_2_ [28]. In this regard, Pt-Ru-based catalysts are found to be excellent catalysts that result in a lower CO oxidation potential of about 0.220 V vs. RHE when compared to 0.325 V on Pt catalysts [29]. Furthermore, compared to pure Pt catalysts, which require a potential of 0.8 V vs. RHE for water breakdown, Ru requires a lower potential of 0.2 V vs. RHE. This helps to capture oxygenated intermediate species for the oxidative removal of adsorbed CO. The classical bifunctional mechanism of the Pt-Ru catalyst is given in Figure 2b. To date, Pt-Ru catalysts are said to be state-of-the-art anode catalysts and are considered the standard to compare the electrocatalytic activity of novel catalyst systems. There have been several advancements in Pt-Ru-based electrocatalysts for enhanced MOR [30].

In addition to the electrochemical MOR activity, stability is also an important factor that affects commercial applications. The Pt/C and Pt-M/C (M = transition metal or noble metal) based catalysts also suffer from poor stability. It is well known that over time, Pt/C and Pt-M/C catalysts undergo degradation due to several degradation mechanisms, such as Pt nanoparticles agglomeration, Pt nanoparticle dissolution and re-deposition, coalescence, and carbon support corrosion. Among these degradation mechanisms, carbon support corrosion is one of the challenging issues with the anode catalysts. The catalyst support, generally the high surface area carbon, serves many different purposes, such as (i) it serves as a platform to disperse nanosized Pt nanoparticles, (ii) electron conducting medium to accelerate the reaction kinetics by electron donation or electron withdrawal, (iii) high surface area helps to enhance the utilization of the noble metal nanoparticles surface, and (iv) porosity that helps the reactants and products in/out on the catalysts surface [31]. The most commonly used support materials are carbon black (Vulcan XC-72R) and Ketjenblack (KB) due to their high surface area and good electronic conductivity [32,33]. However, it is well known that Vulcan carbon is highly sensitive to electrochemical carbon corrosion due to its poor graphitic nature and the presence of various oxygen surface functionalities [34]. In addition, the Vulcan carbon also contains dead-end pores, and the Pt nanoparticles buried inside the dead pores are not accessible to electrochemical reaction, significantly reducing the overall catalyst utilization and making the Vulcan carbons susceptible to potential instability effects [35]. In order to improve the stability and utilization of the noble metal nanoparticle surface of Pt/C and Pt-M/C catalysts, several alternative carbon supports have been developed, such as graphene, N-doped graphene, N-doped carbons, carbon nanotubes, carbon nanofibers, and mesoporous carbons [36]. Although a significant improvement in catalyst utilization and stability has been achieved, a considerable amount of catalyst degradation is still seen, irrespective of the type of carbon supports [37]. In this regard, researchers have also investigated the effect of non-carbon metal oxides as possible support materials [38]. TiO_2_-based non-carbon support is the most common and popular type that exhibits stable performance and anti-corrosion ability in harsh atmospheres such as high acidity [39]. In addition, TiO_2_’s secondary d-electron structure has the potential to strengthen the metal-carrier synergy and improve chemical, electrochemical, and photochemical activity via structural modulation [40]. In addition, the TiO_2_ demonstrated rapid CO oxidation kinetics due to its intrinsic ability to form Ti-OH species upon the water dissociation on its surface, just like Ru does [41]. A variety of non-carbon meal oxides have been explored as possible support materials; however, the MOR performance of non-carbon-based support materials is not satisfactory, due to their low surface area and poor electronic conductivity [42,43].

In this regard, unsupported metal aerogel catalysts have recently become more appealing materials because they can form a thin catalyst layer on their own without the aid of supporting elements such as carbon. The unique porous network of unsupported catalysts offers quicker proton and electron pathways due to direct contact between the metallic active sites and reactants that can enhance the catalyst utilization and electrochemically active surface area for MOR. In addition, the unsupported catalysts endow the advantages of high crystallinity and strong metal-metal interaction that result in enhanced chemical and electrochemical stability against dissolution and agglomeration [44,45,46]. Additionally, because unsupported catalysts do not have carbon supports, which are prone to electrochemical carbon corrosion, they are generally more durable. Additionally, avoiding carbon supports can simplify the synthesis protocol and increase the cost-effectiveness of the one-pot synthesis method. Significant recent efforts in this area have produced adequate advancements in unsupported Pt-based electrocatalysts in terms of composition modulation, morphology tailoring, and mechanism exploration, such as noble metals in the form of 3D open nano frames, nano cubes, nanotubes, core-shell structured catalysts, concave nano cubes and nanoflowers, 1D Pt nanostructures, nanocages, and dendritic alloy nanostructures [47,48,49].

## 3. Metal Aerogels

By definition, a gel is defined as a “non-fluid colloidal network or polymer network that is expanded throughout its whole volume by a fluid”. Therefore, gels are basically originated from the colloidal solutions of particles with a size between 1 µm > colloids > 10 nm dispersed in a continuous liquid phase. The gels are generally obtained from the sol-gel synthesis method followed by a drying process that results in powders that can be used for a variety of energy-related applications [50,51]. A special drying process called freeze-drying or supercritical drying is followed to transform the gels into aerogels. Metal aerogels are fascinating, unsupported, self-assembled, 3D porous nanostructures with ultra-low density and a large specific surface area, and are abundant interconnected porous materials that have been gaining tremendous interest in recent years in heterogeneous catalysis and electrocatalysis [52,53]. Metal aerogels are fully made of metallic atoms connected by metal-metal bonds; they share unique physicochemical properties of traditional aerogels, such as large surface area, porous network, and metals, such as high electronic conductivity and catalytic activity [54]. In this context, metal aerogels (MAs) exhibit unparalleled potential as highly active and durable electrocatalysts, surpassing conventional non-metal and unsupported metallic catalysts, due to their robust structure, rapid mass/electron transfer pathways, and tunable compositions [55].

**Figure 3:**
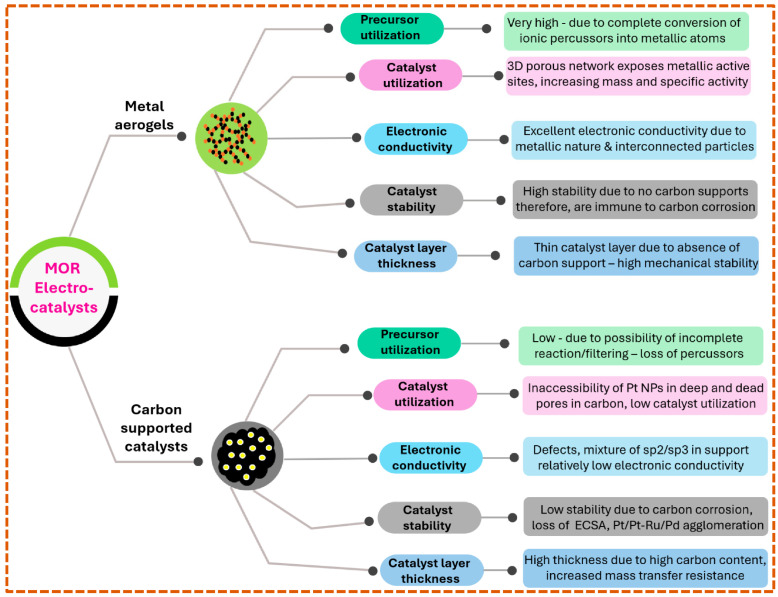
Comparison of metal aerogel catalysts vs. traditional carbon-supported catalysts.

Metal aerogel catalysts possess several advantages over conventional/traditional carbon-supported catalysts (Figure 3) for the methanol oxidation reaction, as shown below.

1. *Stability:* Metal aerogels are made of completely metallic atoms with no carbon supports. Therefore, the catalyst degradation associated with electrochemical carbon corrosion can be completely mitigated with the use of unsupported metallic aerogels. Metal aerogel-based electrocatalysts consequently exhibit excellent stability under electrochemical conditions.
2. *Electronic conductivity:* Compared to traditional carbon supports whose electronic conductivity highly depends on the graphitization content and surface functional groups, metal aerogels contain all the metallic atoms, therefore promoting efficient electron transfer along the nanostructure between the reactants and the catalyst surface. Higher rate of electron transfer enhances the rapid electron movement and hence enhances the MOR kinetics.
3. *Electrochemically active surface area:* The conventional carbon supports, such as Vulcan carbon-supported catalysts, usually suffer from a low electrochemically accessible surface area. This is due to the presence of dead pores in which the deposited metallic nanoparticles are deeply buried and not accessible to electrochemical reactions. In contrast, the unsupported metal aerogel contains a network of metallic atoms connected to each other, with 3D porosity that allows accessibility to every metallic atom and hence results in a higher electrochemically active surface area.
4. *3D porosity and mass transfer:* The conventional carbon support usually suffers from mass transfer issues due to insufficient porosity. In contrast, metal aerogels contain an excellent 3D porous network that allows easier movement of reactant molecules to the metallic active sites.
5. *High mass activity and catalyst utilization:* The conventional carbon-supported catalysts often result in lower mass activity because the accessible noble metals are buried in the carbon, therefore requiring higher catalyst mass loadings. In contrast, due to high catalyst utilization, metal aerogels can produce higher electrocatalytic activity at lower catalyst mass loadings and hence result in several other higher mass activities compared to carbon-based catalysts.
6. *Reduced catalyst layer thickness:* The conventional carbon-supported catalysts contribute to higher catalyst layer thickness on GC and MEAs, which significantly impacts mass transfer issues. This is due to the presence of a large amount of carbon that results in a high catalyst layer thickness. In contrast, the metal aerogel contributes significantly less catalyst thickness due to no carbon support, and hence it is expected to reduce the resistance that comes from a thicker catalyst layer.
7. *Simple synthesis:* Metal aerogel catalysts are synthesized through the reduction of metallic precursors with the assistance of reducing agents, which typically necessitates no additional treatment. In contrast, the traditional impregnation catalyst synthesis processes necessitate heat treatment or special post-synthesis procedures to eliminate ligands, surfactants, polymers, or structural directing agents, which complicate and prolong the synthesis process.
8. *Prospect:* Time efficient, convenient, highly active, and stable catalyst that can be obtained and upscaled to large-scale industrial applications, whereas the carbon-supported catalysts lead to uneven distribution of the metallic active sites on the carbon, along with the complicated, time-consuming synthesis process.

## 4. Synthesis of Metal Aerogels

**Figure 4:**
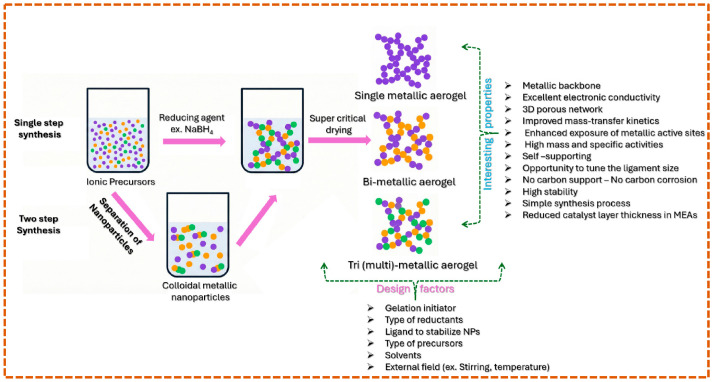
Schematic showing the single-step and two-step synthesis of metal aerogels, interesting properties, and design factors [obtained from Ref. [56], open access].

The conventional sol-gel synthesis process is the most adopted synthesis process for metal aerogels. This consists of dissolving the metal precursors in an aqueous solution, followed by adding reducing agents to obtain the metallic atoms that undergo self-assembly into a hydrogel. The hydrogel is then subjected to a special drying process called supercritical drying/freeze-drying to obtain the aerogel (Figure 4). Further, depending on whether or not the obtained colloidal nanoparticles are separated (purification) before hydrogel formation, the metal aerogel (MA) synthesis techniques are classified into two-step or single-step methods [56].

### 4.1. Two-Step Synthesis Methods

The first noble MA was synthesized by using a two-step synthesis process, which mainly consists of two steps: (a) obtaining colloidal NPs by adding the reducing agents, (b) purification and concentrating the colloidal solution, self-assembly of NPs into a hydrogel by gelation process, followed by supercritical drying to obtain the MAs. A variety of noble MAs have been synthesized by using a two-step synthesis process. However, the recent MAs synthesis process has been shifted to one-step methods. This is due to tedious purification and concentration steps, longer gelation time required for the two-step synthesis process.

### 4.2. One-Step Synthesis Methods

The one-step MA synthesis process is by far the most adopted method due to its simplicity and convenience, which does not require the separation of NPs, essentially integrating NP preparation and gel formation in a single step. When it comes to controlling the proportion and composition of materials, the one-step process is superior to the two-step process; however, it is unable to control the microstructure of aerogels. Both of these approaches have benefits and can be applied to the core-shell, hollow architecture, and alloy combinations that are commonly employed in the electrocatalysis field. With the two-step synthesis process, the gelation time has been greatly reduced from several days to hours and in some cases minutes. Therefore, the one-step synthesis process remains the most popular for MA synthesis.

### 4.3. Mechanism of Metal Aerogel Formation

**Figure 5:**
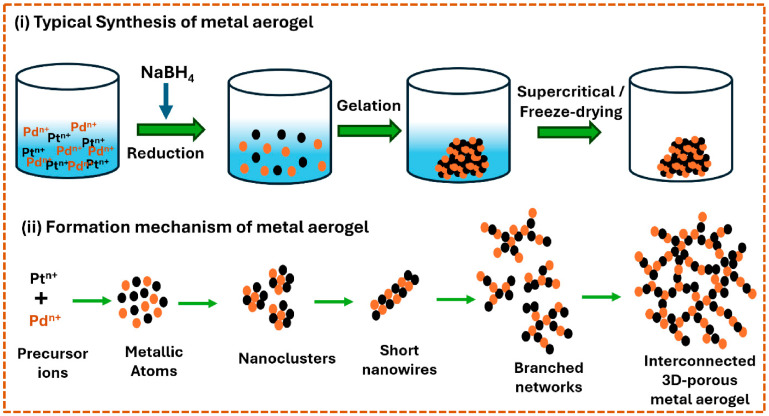
(**i**) Schematic showing the single-step metal aerogels, typically with the NaBH_4_ reduction method, (**ii**) formation mechanism of metal aerogels with an example of Pt-Pd metal aerogel.

The general mechanism of metal aerogel formation includes the formation of metallic, colloidal NPs typically by NaBH_4_ reduction method and the self-assembly of NPs to form a hydrogel, which, upon drying, produces a 3D nanoparticle framework of MAs (Figure 5). The most crucial component of MAs is the gelation process, which creates a network of highly porous, three-dimensionally connected nanoparticles by carefully destabilizing the dispersion. Often, the colloidal stable NPs experience repulsive forces (either by electrostatic repulsion or by steric repulsion if organic ligands are used) to undergo self-assembly, which therefore requires longer gelation times. The forced destabilization is a phenomenon that reduces the repulsive forces and enhances attractive forces to bring the NPs closer to each other to self-assemble into hydrogels [57]. Therefore, the introduction of destabilizing agents has been an effective method to induce gelation and reduce the gelation time significantly. A variety of destabilizing agents have been developed. Finally, the hydrogel undergoes a special drying process called supercritical drying or freeze-drying to obtain the final aerogels. The widely investigated noble MA catalysts have been shown to have 3D interconnecting nanowire structures. According to Wen et al. [58], the nanoparticle fusing and evolution of metal aerogels can be detailed as shown below. The Au hydrogel is formed by inducing the *β*-CD−Au NPs to combine using dopamine as a destabilizing agent. After the addition of the dopamine, gelation was started, and a fluffy black solid containing the Au hydrogel was obtained after 6 h. It was also found that the gelation time can be modified by changing the concentration of dopamine. The produced Au aerogel reveals a web of bifurcated, extremely porous, and linked structures resembling very thin wires. With both meso- and macropores open, their pore size distribution is quite wide. It was observed that host–guest interaction between *β*-CD and dopamine is one of the driving forces for the Au hydrogel formation. By accumulating TEM images at various gelation times, the source of the Au metal aerogel network was determined. It was observed that dopamine induces the fusion of nanowires within 2 h, starting with the assembly of several small NPs, progressing to short nanowires, small, branched networks, and finally into large-scale interconnected nanowire networks. There was no evidence of aggregation after 2 days, and what was visible were densely linked Au nanowires of about 5–6 nm. When the β-CD was replaced by other stabilizers, such as pyrocatechol, 2-phenylethylamine hydrochloride, ferrocene carboxylic acid, and citrate, different Au morphologies were obtained. Based on the above discussion, it is clear that the colloidal NPs self-assemble into short nanowires, small, branched networks, and finally large-scale interconnected nanowire networks as a possible and widely accepted mechanism of MA formation with a 3D-porous, interconnected nanowire network (Figure 5). MA synthesis, morphology, and composition depend on several factors, the most important of which are (a) initiators, (b) precursors, (c) reductants, (d) ligands, (e) solvents, and (f) external fields. A detailed gelation mechanism and the design strategies of metal aerogels are omitted in this review, and the readers are advised to refer to the following excellent reviews by the author and Prof. Alexander Eychmüller, an eminent scientist in the field of metal aerogels [59,60,61,62,63,64,65].

**Figure 6:**
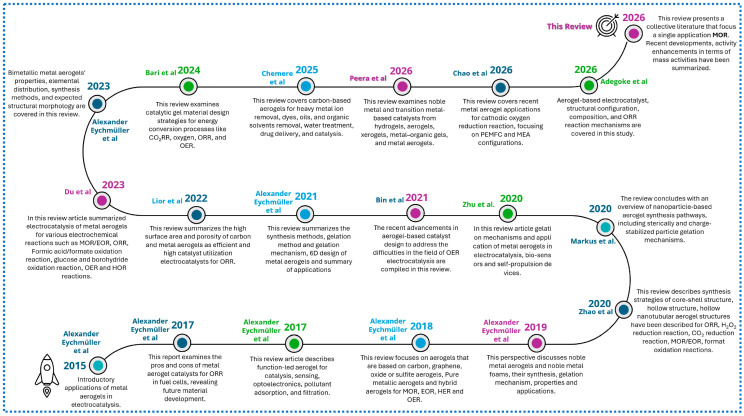
Timeline showing different reviews that have been published on gel-based catalysts since 2015–2026 Alexander Eychmüller et al. (2015) [62], Alexander Eychmüller et al. (2017) [66], Alexander Eychmüller et al. (2017) [67], Alexander Eychmüller et al. (2018) [68], Alexander Eychmüller et al. (2019) [61], Zhao et al. (2020) [65], Markus et al. (2020) [63], Zhu et al. (2020) [59], Bin et al. (2021) [69], Alexander Eychmüller et al. (2021) [60], Lior et al. (2022) [53], Du et al. (2023) [55], Alexander Eychmüller et al. (2023) [64], Bari et al. (2024) [70], Chemere et al. (2025) [71], Peera et al. (2026) [72], Chao et al. (2026) [73], Adegoke et al. (2026) [74].

We have noticed that 18 review articles have been published on aerogels and metal aerogels since their first discovery in 2009. In common with several review articles, we introduce metal aerogels and describe the synthesis routes, properties, gelation mechanism, and morphological control of metal aerogels [53,55,56,59,60,61,62,63,64,66,67,68,69,70,71,72,73,74]. Almost all the reviews seem to be very much overlapping with repetitive content. Moreover, the 18 review articles that we examined describe mostly ORR, oxygen evolution reaction, ethanol oxidation reaction, and other mixed applications, with no focus on a specific application, especially MOR. Reviews with mixed applications usually give an overall view of metal aerogels (Figure 6). However, researchers in a particular field need a summary of important advancements altogether in one place to understand the research gaps that provide opportunities to synthesize efficient catalysts. Specifically, a summarized review of the metal aerogel applications of MOR, to the best of our knowledge, has hardly ever been reported in the past decade. This review presents a collective literature that focuses on a single application of MOR. Recent developments and activity enhancements in terms of mass activities have been summarized.

## 5. Metal Aerogel Catalysts for MOR

### 5.1. Pt and Pt-Alloy Metal Aerogels

**Figure 7:**
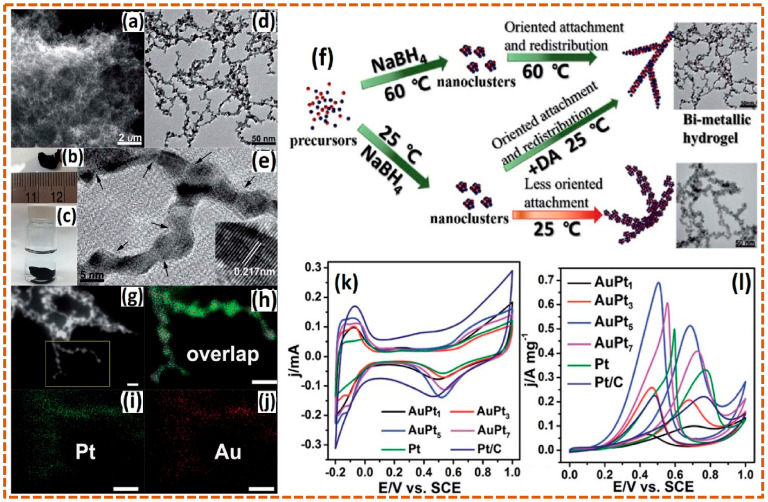
(**a**) SEM, (**b**) picture of solid catalysts, (**c**) picture of hydrogel, (**d**,**e**) TEM image of AuPt_5_ metallic aerogels, (**f**) schematic representation of AuPt_5_ metallic aerogels, (**g**–**j**) TEM elemental mapping of AuPt_5_ metallic aerogels, and (**k**,**l**) cyclic voltammetry curves of AuPt_5_ metallic aerogels of different Au and Pt compositions along with Pt/C (1.0 M H_2_SO_4_/1.0 M H_2_SO_4_ + 1.0 M CH_3_OH) [reproduced after permission from Ref. [81]].

Metal aerogels that combine the properties of metals and aerogels are interesting materials for electrocatalysis due to their highly interconnected metallic network and hierarchical porous structure that enhances the mass transport issues and improves the stability of the catalysts [75]. With these characteristics, metallic aerogels are a promising material for the next generation of electrocatalysts that will power the energy revolution. In addition to the advantages of structural features, the electrocatalytic activity of metal aerogels also depends on surface coordination active sites on the metallic network. These active sites include uncoordinated atoms, which are defective sites that significantly affect the binding energy of the reaction intermediates [76,77,78]. Shuai Lin et al. proposed a dendritic Pt aerogel (DPAs) synthesized via a sol-gel process. The coordination environment, defects, and surface-active sites significantly influence the reactants and their adsorption behavior in electrocatalysis. Specifically, the edge, corner, and defect atoms and defect sites offer potential catalytic active sites for electrochemical reactions [79,80]. In addition, the molecular and kinetic control of the gelation process is paramount for realizing the faster synthesis process from a commercial point of view. Furthermore, surfactant and ligand-free synthesis strategies are important as there is a possibility of ligand adsorption, requiring special post-synthesis steps, which not only complicate the synthesis process but also negatively affect the electrocatalytic activity. In this regard, Shi et al. [81] synthesized a surfactant-free metal aerogel of AuPt_5_ that exhibits excellent electrochemical activities for methanol electrooxidation (Figure 7a–l). The AuPt hydrogel was synthesized by using NabH_4_ reduction at 60 °C, followed by the addition of dopamine as a destabilizing agent. The AuPt metal aerogel was obtained from supercritical drying of the AuPt hydrogel that exhibited a unique 3D network of AuPt particles interconnecting with each other with excellent porosity. The elemental mapping of the AuPt aerogel was seen to have Au and Pt in close proximity, suggesting an alloy behavior. A detailed formation mechanism of the AuPt_5_ 3D-metal aerogel is followed by recording the TEM images of the precursors at different time intervals. The mechanism of formation of interlinked nanowires is ascribed to (i) nanoparticle formation into clusters, (ii) fusing of nanoclusters, (iii) generation of short nanowires, and (iv) growing into a 3D network. The proposed mechanism is quite similar to the mechanism proposed by Prof. Alexander Eychmüller in his first discovery of Au and Pd metal aerogels [82]. Therefore, it is accepted as a general and universal mechanism of metal aerogel formation. In addition, the authors also investigated a strategy to kinetically control the synthesis of metallic AuPt hydrogels via (i) increasing the temperature, and (ii) adding DA (dopamine) at room temperature. Between these two, elevating the temperature would be more reasonable in terms of enhancing the surface energy of nanoclusters, leading to the rapid attachment into nanowires [83].

On the other hand, the addition of dopamine leads to the generation of interlinked nanowire network structures with isotropic attachment to nanoclusters with smaller nanowire diameters. Therefore, a combined effect of temperature and the addition of dopamine leads to a robust and versatile approach for accelerating the gelation and controlled orientation of nanochains in the metal aerogels. However, the complete removal of surface dopamine is difficult, and it comes with certain compromises in the MOR activity. The electrochemical studies reveal that AtPt_5_ metal aerogel catalysts possess a higher ECSA of 53.9 compared to 40.1 for Pt metal aerogel and show enhanced methanol oxidation peaks, onset, and mass activities, suggesting that AuPt_5_ possesses excellent MOR activity. When compared to AuPt_5_ metal aerogel, the AuPt_5_-25-DA showed 35% less mass activity compared to AuPt_5_ synthesized at 60 °C. This is attributed to the significant blockage of DA via strong adsorption and disruption in the electron transfer during MOR.

In another work, Tie et al. [84] synthesized a 3D porous PtCu aerogel by using NaBH_4_ without any surfactants or ligands as destabilizers. Cu is chosen as an oxophillic metal to improve the intrinsic catalytic activity by changing the electronic environment on the surface of Pt through the strain effect for MOR. A single-step synthesis of PtCu aerogel was achieved in about 8 h of gelation time with an R/M ratio of 1/100. The PtCu aerogel morphological analysis reveals the well-interconnected porous self-supporting architectures connected by thin nanowires. It is believed that highly porous morphology improves the utilization of metallic active sites and enhances the easier diffusion of methanol molecules, contributing to the enhanced methanol oxidation currents. The Pt_3_Cu_1_ TEM observations reveal lattice boundaries and defects along the interconnected nanowires, which is considered to be helpful to the catalytic activity [85]. The XPS analysis reveals that Pt exists in Pt^0^/Pt^2+^ oxidation states, along with Cu in the form of Cu^0^/Cu^2+^ oxidation states, with metallic Pt^0^ and Co^0^ as the most dominant species for MOR. In addition, the positive shift in the Pt peaks in Pt_3_Cu_1_ relative to Pt aerogel indicates that electrons are transferred from Cu to the Pt atom [86]. One possible explanation is that the shift in Pt binding energy pushes the d-band center downward, which may facilitate the desorption of CO intermediates and oxygen-containing molecules from the catalyst’s surface [87]. Furthermore, due to Cu’s higher oxygen affinity on the Pt aerogel surface, it can enhance the catalytic oxidation of methanol by promoting the breaking of O-H bonds in H_2_O molecules, leading to the generation of more OH_ads_ that help produce a faster anti-CO poisoning effect [88]. Based on the electronic interactions from the XPS analysis, it is assumed that Pt_3_Cu_1_ could enhance the MOR activity compared to Pt aerogel. This assumption was verified by recording the cyclic voltammetric measurements in 0.5 M H_2_SO_4_ + 1 M CH_3_OH. It is seen that Pt_3_Cu_1_ aerogel catalyst exhibits higher MOR activity compared to Pt aerogel and commercial Pt/C catalyst, with a higher I_f_/I_b_ of 1.22 compared to 0.89 and 1.03 for Pt aerogel and Pt/C catalyst, indicating that Pt_3_Cu_1_ aerogel exhibits higher MOR activity. In addition, the mass activities of Pt_3_Cu_1_ are found to be 6.56 times higher than the counterparts, indicating that the Pt_3_Cu_1_ aerogel catalyst has high exposure of active sites resulting from the extended porous network. In addition to the excellent mass activity, the Pt_3_Cu_1_ also exhibited excellent stability, as assessed via TEM analysis after 1000 cycles in 0.5 M H_2_SO_4_ + 1 M CH_3_OH, where the 3D self-supporting architectures of Pt_3_Cu_1_ aerogel remain intact, indicating that Pt_3_Cu_1_ aerogel has excellent stability.

**Figure 8:**
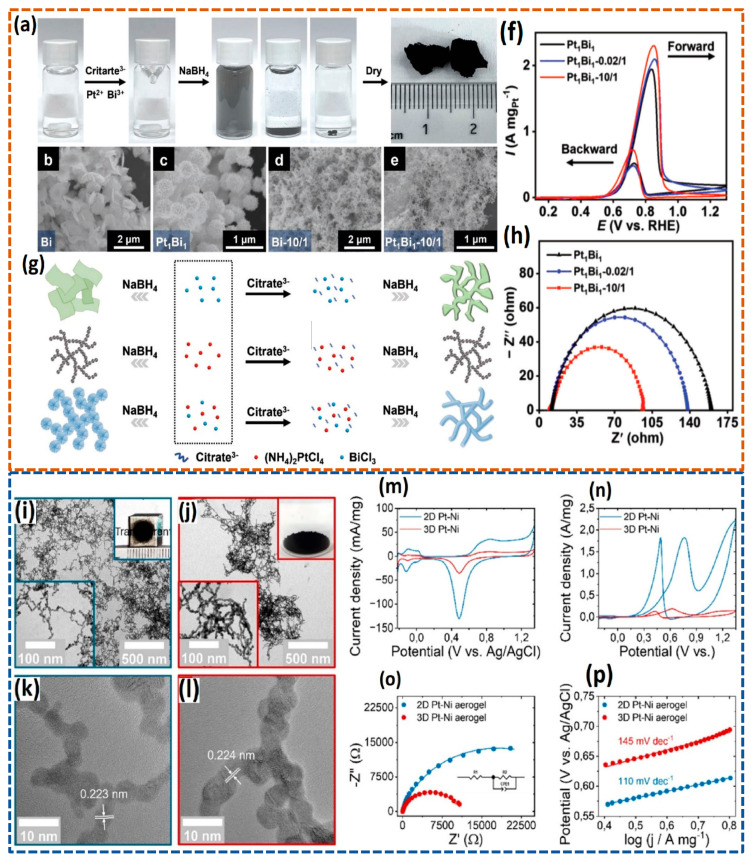
(**a**) The Pt_1_Bi_1_-10/1 aerogel fabrication process captured in photographs, (**b**–**e**) scanning electron micrographs illustrating the composition-directed control of the aerogels’ NBBs, (**f**) cyclic voltammetry curves of various Pt_1_Bi_1_ metal aerogel catalysts, (**g**) a schematic of the manufacturing procedure, and (**h**) EIS spectra of various Pt_1_Bi_1_ metal aerogel catalysts. 1.0 M KOH + 1.0 M CH_3_OH electrolyte [reproduced with permission from Ref. [93]]. (**i**–**l**) TEM images of 2D and 3D Pt-Ni aerogel at different magnifications, respectively, (**m**,**n**) CV curves normalized with mass of 2D and 3D Pt-Ni metal aerogels in 0.5 M H_2_SO_4_/0.5 H_2_SO_4_ + 0.5 M CH_3_OH, respectively, (**o**) EIS spectra, and (**p**) tafel slopes of 2D and 3D Pt-Ni metal aerogels [[94], open access].

Largely, the MAs are synthesized by using metal slats as percussors and NaBH_4_ as reductants via a sol-gel synthesis process, and the MAs synthesized via this method generally lead to 3D-nanowire network morphologies. However, optimizing performance and conducting thorough investigations into structure-property correlations are both severely limited when dealing solely with nanowire morphologies. In order to address this, several strategies have been proposed, such as post-treating the as-synthesized metal aerogels that could yield nanotubular structure aerogels [85,89,90], by using CO gas as reductant and coordinating agent via the “adsorption-decomposition” growth model [91], manipulating the nucleation step during the reduction process by altering the precursor order of the reactants to yield core-shell structured bimetallic aerogels [92]. However, most of these methods are time-consuming and also involve the handling of toxic agents such as CO, demanding easier and sustainable synthesis routes to the metal aerogels of diverse morphologies. In such an attempt, Xue et al. [93] proposed a sodium citrate ligand-based sustainable and low-cost synthesis route to fabricating MAs of diverse morphologies with 0D, 1D, 2D, and 3D. A unique photo-electrocatalyst was prepared by carefully manipulating the optical/electrocatalytic activity of the MAs via a semiconducting Bi and conducting Pt as active sites (Pt_1_Bi_2_). Without citrate as ligands, the Bi aerogel exhibited a sheet morphology, and Pt showed a nanowire morphology, whereas Pt-Bi showed a 3D microsphere with a Pt% of more than 25% in the composition. Furthermore, with increasing Pt composition, 3D microsphere morphology changes from loosely compacted to closely compacted with increasing Pt proportion from 33.3% for Pt_1_Bi_2_ to 75.0% for Pt_3_Bi_1_. With citrate as ligand, a distinct morphology has been observed for Pt_x_Bi_y_ in the aerogel; furthermore, increasing the L/M ratio was also found to affect the morphology. Increasing L/M from 0 to 10 causes the Bi system’s 2D nanosheets to become irregular matter, and the Pt_1_Bi_1_ system’s 3D microspheres to become 1DNWs (Figure 8a–g). While assessing the Pt_x_Bi_y_, the *I_F_* of the methanol oxidation current correlates to the Pt content, meaning, with increasing Pt content, the *I_F_* increases from 1.80 A mg_Pt_^−1^ for the Pt_1_Bi_2_ aerogel to 3.05 A mg_Pt_^−1^ for the Pt_2_Bi_1_ aerogel, which are found to be higher than the Pt/C catalysts with a mass activity of 0.70 A mg_Pt_^−1^, and are attributed to the increasing conducting nature of the electrocatalysts due to Pt. On the other hand, the structural effect of Pt_x_Bi_y_ attained from the different L/M ratio also exhibited morphology-dependent MOR activity. For *I_f_* of Pt_x_Bi_y_ catalysts with a L/M ratio of 0.02/1 and 10/1, the mass activity increases from 2.09 to 2.30 A mg_Pt_^−1^ (Figure 8f,h). This is attributed to the morphological distinction of metal aerogels, in which the electronic movement along the microspherical-based morphology of Pt_1_Bi_1_ aerogel is harder than the 1D nanowires structured metal aerogels for the Pt_1_Bi_1_-10/1 catalyst. Furthermore, the combined 1D nanowires and core-shell morphology of the Pt_1_Bi_1_-10/1 catalyst can largely expose catalytically active Pt sites, further promoting the performance. This study concludes the importance of metal aerogels for the enhanced electrical conductivity attributed to the 1D nanowire morphology.

Metal aerogel synthesis has been considered expensive due to the fact that it requires high concentrations of noble precursors that incur high production costs. Furthermore, the high cost associated with the supercritical drying of the gel that requires CO_2_ gas or liquid N_2_ necessitates research into more cost-effective drying alternatives. For the large-scale production of metal aerogels, it is important to solve these problems. In an attempt to overcome the above challenges, recently, Pavel Khavlyuk et al. [94] reported a reproducible methodology to synthesize Pt-Ni aerogel via the phase-boundary-gelation method, which requires less precursor quantity and does not need expensive drying techniques [95]. The unique stamping method has been developed in which a previously obtained aqueous Pt-Ni nanoparticle solution has been placed onto a cover glass that has high surface tension. An organic solvent mixture (toluene + ethanol) is then added to the solution. This creates an aqueous-non-aqueous phase boundary. At the boundary, the gelation initiates, and in parallel, the low-boiling-point organic solvents evaporate, leaving behind a 2D network of aerogel floats on the aqueous surface. This process was carried out on the surface of the GC repeatedly until an 8-layer catalyst was coated on the surface. One uniqueness of this method is that the metal aerogel did not undergo any supercritical drying step. This process was compared with the 3D Pt-Ni aerogel catalyst that was synthesized via a traditional supercritical drying process. Surprisingly, the 2D Pt-Ni aerogel synthesized via phase-boundary gelation strategy exhibited a mesh-like structure, very much similar to the 3D Pt-Ni aerogel catalyst (Figure 8i–l). Specifically, the 2D Pt-Ni aerogel displayed more complex branched structures compared to the 3D Pt-Ni aerogel, which had a more uniform distribution of elements throughout the network, with Ni being more abundant in the center and Pt being more abundant on the ligament surfaces. Surprisingly, the 2D Pt-Ni exhibited very high methanol oxidation currents compared to 3D Pt-Ni. Though the enhanced MOR can be attributed to the modification of the d-band electronic structure of Pt via Ni, and the ability of Ni to provide more oxygen-containing species at lower potentials facilitates easier oxidation of adsorbed molecules. This results in enhanced mass activity of 1.8 A mg^−1^ for Pt-Ni aerogel, in contrast to 0.2 A mg^−1^ for 3D aerogel (Figure 8m–p). In order to lower the cost of the catalyst and simplify the synthesis process, this study shows that there are viable alternatives to the conventional supercritical drying of metal aerogels.

**Figure 9:**
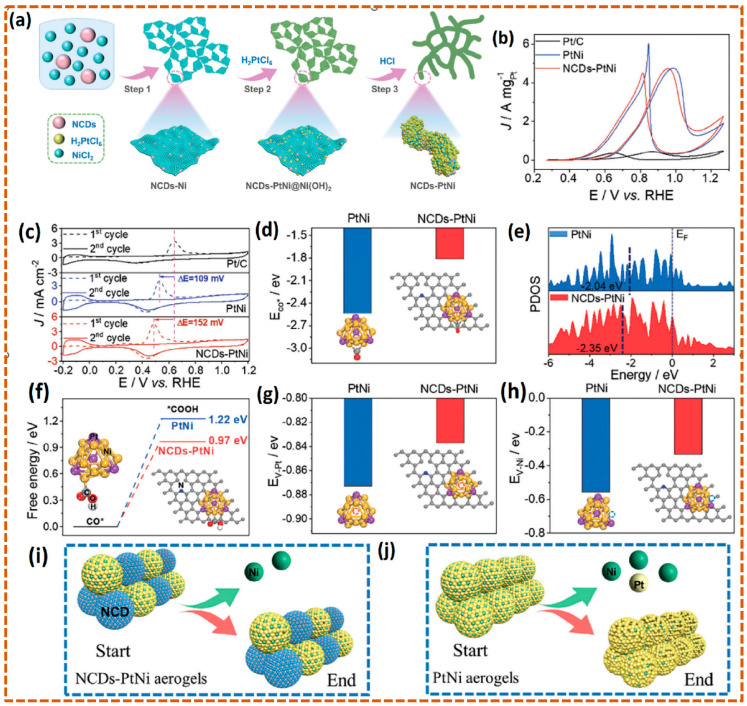
(**a**) Schematic diagram for the synthetic process of NCDs-Pt-Ni aerogels. CV curves (**b**) in methanol solutions of Pt/C, Pt-Ni, NCDs-PtNi aerogel in 0.1 M HClO_4_ + 1.0 M CH_3_OH, (**c**) CO-stripping CV curves of Pt/C, Pt-Ni and NCDs-Pt-Ni aerogel in 0.1 m HClO_4_ solution, (**d**) adsorption energy of *CO intermediate (E_CO*_), (**e**) PDOS of Pt atoms in Pt-Ni aerogel and NCDs-Pt-Ni aerogel, (**f**) free energy diagram of *CO oxidized to *COOH on Pt-Ni and NCDs-Pt-Ni aerogel, (**g**) EV-Pt, and (**h**) EV-Ni for Pt-Ni and NCDs-Pt-Ni; schematic diagram of surfaces changes on (**i**) Pt-Ni and (**j**) NCDs-Pt-Ni aerogel during acidic MOR electrocatalysis [reproduced with permission from Ref. [98]].

Although the metal aerogel electrocatalysts possess excellent electronic conductivity due to a continuous metallic network, researchers still believe that metallic aerogels suffer from problems such as metal aggregation and deactivation. Nevertheless, incorporating the metal aerogels on carbon supports might solve the problem to some extent; the addition of carbon supports to make composite materials could lead to the disappearance of the advantages of metal aerogels. In addition, weak interaction between two composite materials might decrease the overall performance [96,97]. Therefore, it was believed that integrating the nanosized, low-dimensional carbon materials into the metallic aerogel nanochain networks gained special interest. Integrating carbon materials is believed to enable the interfacial interaction between the metal and carbon atoms to enhance stability and mitigate the metal nanoparticle aggregation. In this regard, Li et al. [98] proposed N-doped carbon dots integrating into the metal aerogels (NCD-PtNi) to anchor metal atoms and protect them from dissolution and aggregation. Aerogels made of NCDs and PtNi display a number of desirable properties, including 3D hierarchical porous morphology, an alloyed PtNi structure, and robust NCD-PtNi interaction (Figure 9a). Experimental characterizations and theoretical analyses suggest that these features contribute to more active sites, better resistance to toxic intermediates, and prevention of metallic atom dissolution and aggregation during electrocatalysis. In a typical synthesis, pre-synthesized quantum dots have been mixed with Ni precursors to form NCDs-Ni nanosheet aerogels to which the Pt precursors are added and fast reduced to form the NCDs-PtNi@Ni(OH)_2_. The Ni(OH)_2_ was dissolved with the help of HCl, resulting in the final product of NCDs-PtNi aerogels. The control experiments suggest that PtNi and NCD-PtNi aerogels both have 3D hierarchical porous morphology, indicating that there is no significant effect of CD incorporation on the PtNi aerogel. The microstructure of NCDs-PtNi aerogels shows more uniform nanochains compared to PtNi aerogels. TEM elemental mapping and XPS analysis confirm the presence of C and N species on the PtNi aerogel. When applied as an anode catalyst, the NCDs-PtNi aerogel catalyst showed 12 times higher mass and specific activities for methanol oxidation compared to commercial Pt/C and PtNi aerogel catalysts, confirming that the incorporation of NCDs enhances the MOR activity (Figure 9b). Most importantly, the NCDs-PtNi aerogels also exhibited excellent stability. The stability of NCDs-PtNi aerogels was assessed by CV curves in 0.1 M HClO_4_ and then assessed by MOR activity for 5000 potential cycles.

Under these conditions, the Pt/C and PtNi aerogels lost higher proportions of ECSA than the NCDs-PtNi aerogels. MAs calculated after 5000 potential cycles show that NCDs-PtNi aerogels retain about 52% of their initial MOR activity, whereas PtNi and Pt/C could only retain the 10 and 15% of their initial activity, clearly confirming that the presence of NCDs significantly mitigates the dissolution and agglomeration of the NCDs-PtNi aerogel catalyst. The mechanism enhanced the stability of the NCDs-PtNi aerogel catalyst. The enhanced stability of NCDs-PtNi aerogels was also due to the lower potential required for *CO oxidation. Negative shifts of 109 and 152 mV for the *CO oxidation potentials were observed in PtNi and NCDs-PtNi aerogels, respectively, when compared to Pt/C. This indicates that more *OH species were formed at lower potentials, which facilitated the *CO oxidation process. The experimental evidence for lower *CO oxidation potentials was also ratified by DFT calculations and a lower d-band center for PDOS charges analysis. The stability analysis of NCDs-PtNi aerogels was analyzed by assessing the vacancy formation energy (E*_v_*) for both Pt and Ni species [99]. It was found that the E*_v_* values for NCDs-PtNi aerogels were much higher than those of the PtNi aerogel, suggesting that it is harder for Pt and Ni atoms to dissolve from NCDs-PtNi catalyst [100]. This clearly indicates that Pt and Ni atoms in PtNi aerogels are more likely to separate from the aerogel skeleton, in contrast to NCDs-PtNi aerogels, which maintained a more intact surface and had fewer Ni atoms detached (Figure 9c–j). This work opens up a new opportunity in the structural modification of metal aerogels for enhancing the MOR activity and stability.

### 5.2. Pd and Pd-Alloy Metal Aerogels

It is well known that the electrocatalytic activities of metal nanoparticles depend on shape, size, morphology, and chemical composition [101]. Furthermore, the interfacial engineering between the metal nanoparticles and the reactants via organic ligands further enhances the charge transfer and hence the electrochemical activity. For example, the polyallylamine functionalized Pt nanoparticles displaced enhanced ORR due to enhanced interfacial electron transfer between the O_2_ and Pt [102]. Gelation is assisted by integrating metal aerogels and the organic ligand, and the simultaneous enhancement in interfacial charge transfer can boost the electrochemical activity of metal aerogels. Wang et al. [103] proposed a unique strategy of synthesizing PtCu metal aerogel via ionic liquid (ILs), aiming to tackle faster gelation of metal nanoparticles and interfacial charge transfer during MOR. ILs are known for their utilization as a functionalizing agent due to their properties, such as their electron-conducting nature and hydrophilicity. In addition, the low surface tension and slow Ostwald ripening of ILs result in high nucleation rates that allow the formation of smaller-sized nanoparticles [104]. The addition of ILs to PdCu nanoparticles was found to reduce the gelation time to 1 h, and the IL was found to attach to the surface of PdCu nanoparticles via electrostatic force. After the supercritical drying, the PtCu metal aerogel was found to have a 3D porous scaffold with an intensive network of nanowire structure with many branches. According to DLVO theory, the electrostatic attraction can be induced by adding a high concentration of ions [105]. Similarly, in this study, the addition of imidazolium ILs, which are composed of imidazolium cations and inorganic anions, acts as salts and therefore induces the gelation and consequently formation of hydrogel at a temperature of 60 °C. The resulting IL/Pd_3_Cu_1_ exhibited enhanced ECSA and MOR activity, attributed to the alloying effect of Cu in the IL/Pd_3_Cu_1_ catalyst, and in the DMFC full cell, the IL/Pd_3_Cu_1_ catalyst delivered a power density of 8 mW cm^−2^.

**Figure 10:**
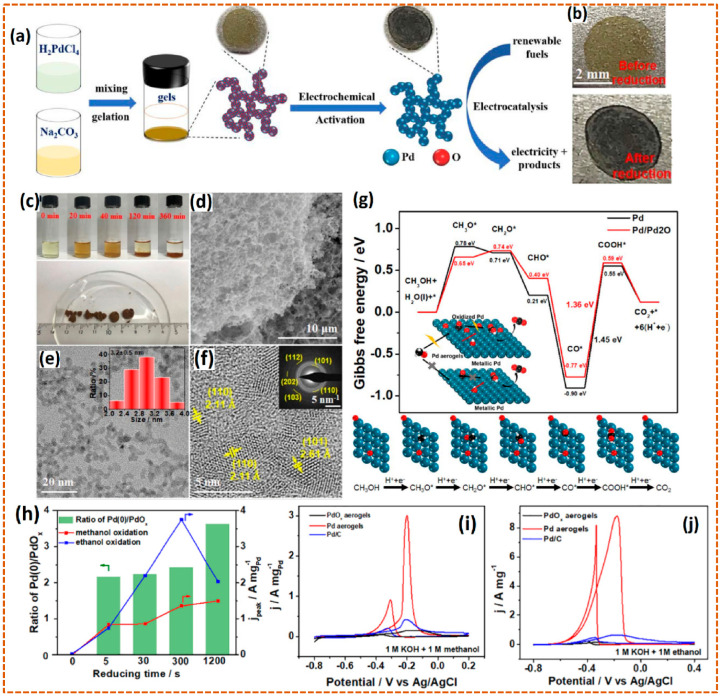
(**a**) The graphical representation of Pd/PdOx catalyst synthesis. Digital images of PdOx aerogels before and after (**b**) electrochemical reduction and (**c**) supercritical drying. (**d**) SEM, (**e**,**f**) TEM images, (**g**) Gibbs free energy and MOR pathway on Pd/PdOx aerogel catalyst, (**h**) histogram showing the ration of Pd/PdOx over reducing times and their relationship with mass activity, and (**i**,**j**) MOR and EOR performance of Pd/PdO_x_ catalysts in 1 M KOH + 1.0 M CH_3_OH/C_2_H_5_OH [obtained from Ref. [106], open access].

In another study, Wang et al. [106] proposed a facile in situ electrochemical method for the synthesis of Pd aerogel catalysts. In this work, a pre-synthesized PdO_x_ hydrogel was obtained via inorganic salt-assisted hydrogelation (Na_2_CO_3_). The PdO_x_ is coated on a Ti plate, and the electrochemical reduction of PdO_x_ to Pd was achieved via the chronoamperometry method at the potential of −1.0 V vs. Ag/AgCl in 1 M KOH electrolyte in a three-electrode system (Figure 10a–g). The electrochemical reduction in Pd was confirmed by the crystalline peaks in the XRD profile and the lattice fringes via TEM measurements. The in situ electrochemical method was found to be convenient and faster than the traditional chemical reduction process. The electrochemically obtained Pd aerogel was found to exhibit excellent MOR activity with a mass activity of 2.99 A mg_Pd_^−1,^ which is 7.12 times higher than the Pd/C catalysts, confirming that electrochemical reduction could be an alternative choice for obtaining metal aerogels (Figure 10h–j). Pd-based materials have been best known for MOR due to their oxidation mechanisms very similar to those of the Pt/C catalyst. However, in the in situ-obtained Pd metal aerogel, it was found to have a mixed phase of metallic Pd and Pd-O_x,_ which might lead to a unique MOR mechanism. DFT studies indicate via partial density of states (PDOS), the d-band center shifts downwards with increasing the oxide surface when compared to Bulk Pd. The increased oxide surface on Pd was thought to weaken the *CO adsorption. A high Gibbs free energy was noticed for the Pd_8_O surface, whereas for the Pd and Pd/PdO_2_ surfaces, a much lower energy barrier was noticed. Therefore, the Pd/Pd_2_O surface is more favored for the better dehydrogenation of methanol to initiate the oxidation process. Furthermore, the adsorption of *CO leads to poisoning of the surface, and the oxidation of adsorbed *CO with the help of *OH is paramount [107]. When compared, the Pd catalyst shows very high *CO adsorption energy, suggesting a strong adsorption, whereas for Pd/PdO_2_, the *CO adsorption was found to be much lower, suggesting that Pd/PdO_x_ possesses a benefit from the anti-*CO poisoning ability. Pure Pd had a larger energy barrier of 1.45 eV, while that for Pd/Pd_2_O was 1.36 eV, suggesting the easier oxidation of *CO on the hybrid surface for Pd aerogels (Figure 10g). Therefore, it can be concluded that in situ reduction of metal hydrogel via the electrochemical method could be one of the alternatives to the traditional chemical reduction methods to obtain the metal aerogels. A similar electrochemical method was also proposed by Yao et al. [108] using Pd and Co to obtain the Pd_3_-(CoO_x_)_1_, which exhibited excellent electrocatalytic activities for MOR, with the mass activities of 4.9 A mg_Pd_^−1^, which is 10.5 times higher than the Pd/C catalyst.

Engineering the electrocatalysts based on orbital coupling has been recently reviewed as an effective strategy to optimize the electrocatalytic activity and stability of electrocatalysts [109]. Specifically, hybridizing the p−d orbitals was found to be an effective strategy to improve the *CO anti-poisoning of Pd-based electrocatalysts for MOR [110,111]. Several p−d hybridization systems were employed, such as Te-Pd, Sn-Ru, and Ga-Ru for alcohol oxidation reactions [112,113]. In this regard, particularly Sn-based catalyst systems have found interesting properties in the Sn-Ru system. It was found that the oxophillic element, such as Sn, modifies the electronic nature of Ru and adjusts the d-band center and promotes the acceleration of *CO removal from the Ru sites [114,115]. Based on this conclusion, Guan et al. [116] developed Pd_3_Sn aerogel catalysts via p-d orbital hybridization for alcohol oxidation reactions that include methanol, ethanol, and glycerol oxidation reactions. The Pd_3_Sn metal aerogel was synthesized using ionic liquid-[APMIm]Br (as the initiator) and the hydrazine reduction method. To the aqueous solution of Pd, Sn precursors, and ionic liquid, hydrazine was added to reduce the metal ions, and the solution was allowed to rest until the hydrogel sank to the bottom of the flask. The solution was then vacuum dried at 60 °C to obtain the aerogel. No freeze-drying or supercritical drying was employed. The ionic liquid-assisted Pd_3_Sn metal aerogel showed excellent crystallinity and morphology very similar to the freeze-drying metal aerogel catalysts. HR-TEM measurements specifically reveal a high density of defects and unsaturated surface atomic sites that act as active sites for reactant adsorption. Furthermore, the Pd_3_Sn exhibited intermetallic behavior with a specific surface area of ~190 m^2^ g^−1^. When applied Pd_3_Sn as anodic electrocatalyst for methanol, ethanol, and glycerol oxidation reactions, the Pd_3_Sn catalyst exhibited enhanced oxidation current for all three organic molecules, owing to the advantages of 3D porous morphology, high crystallinity with large amounts of defects and unsaturated surface atoms, together with p-d hybridization of orbital coupling. Further to the credit of Sn, an oxophilic element, CO-stripping experiments reveal lower *CO oxidation potential for Pd_3_Sn than the Pd metal aerogel and Pd/C, clearly suggesting that Sn effectively reduces the *CO oxidation potentials.

### 5.3. Ternary Metallic Hybrid Aerogels

**Figure 11:**
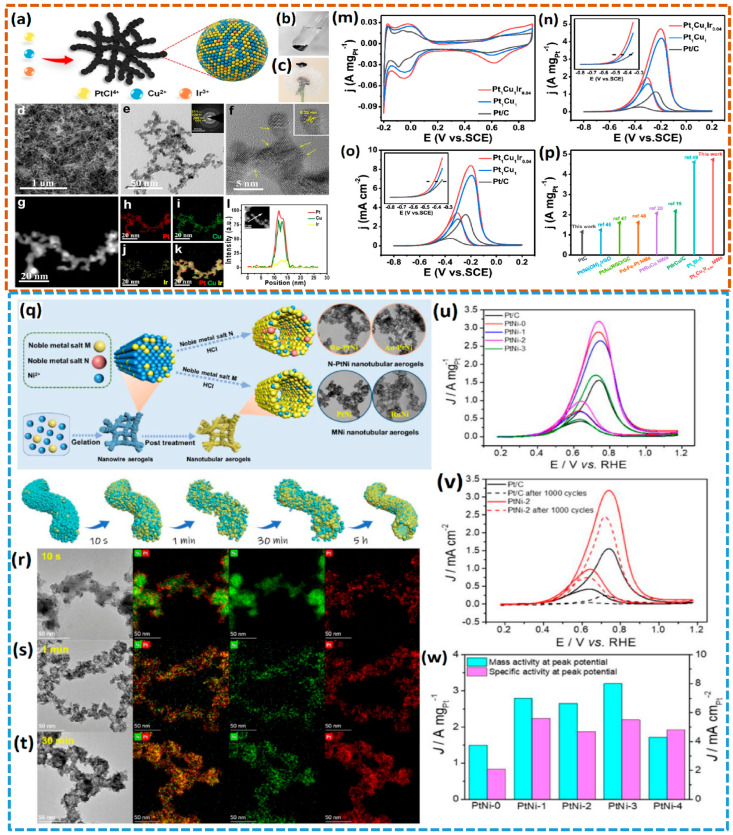
(**a**) graphical representation of Pt_1_Cu_1_Ir_0.04_ catalyst preparation, digital images of (**b**) hydrogel in the test tube, (**c**) dried aerogel on the dandelion, (**d**) SEM, (**e**–**g**) TEM images, (**h**–**l**) TEM elemental mapping of Pt, Cu, Ir, (**l**) EDAX scan profile of Pt_1_Cu_1_Ir_0.04_ catalyst, (**m**) CV curves of Pt_1_Cu_1_Ir_0.04_ catalyst in 0.1 M HClO_4_, (**n**) CV curves of Pt_1_Cu_1_Ir_0.04_ catalyst in 0.1 M HClO_4_ + 1 M CH_3_OH (inset: showing the onset of MOR), (**o**) ECSA-normalized CV curves of various Pt_1_Cu_1_Ir_0.04_ catalysts, (**p**) comparison of mass activities of various catalysts vs. Pt_1_Cu_1_Ir_0.04_ catalysts (118). (**q**) schematic showing the catalyst synthesis process (**r**–**t**) evolution and growing pathway of solid-nanotubular PtNi catalysts at 10 s, 1 min, and 30 min, respectively (the arrows indicates evolution of the nanotubular structures at different time intervals of 10s, 1 min, 30 min and 5 h). Electrochemical tests for the MOR. (**u**) CV curves of various electrocatalysts, (**v**) stability of PtNi-2 aerogels and Pt/C after 1000 cycles, and (**w**) the mass activity of Pt/C and PtNi-x aerogels [120].

Metal aerogels have emerged as a highly promising class of electrocatalysts for MOR because they integrate the intrinsic activity of noble metal nanostructures with a three-dimensional, self-supported, and highly porous framework. In comparison with conventional carbon-supported nanoparticles, aerogels provide continuous conductive pathways, open pore channels, and a high density of exposed active sites, which together favor faster charge transfer, easier reactant diffusion, and better removal of poisoning intermediates during MOR. Recent research has focused on a few key design directions: morphology engineering, dealloying, ternary alloying, trace-element doping, and defect engineering. Wang et al. [117] synthesized ternary PtRuCu aerogels through a one-step in situ reduction process, with gelation completed within 2 h. The optimized Pt_4_Ru_1_Cu_5_ aerogel exhibited a mass activity of 2.07 A mgN^−1^ and a specific activity of 4.1 mA cm^−2^, corresponding to roughly 3-fold and 4-fold improvements over commercial Pt/C, respectively. The performance enhancement was attributed to the synergistic contribution of the porous nanowire network and the ternary alloy composition. In this system, Ru promotes the formation of oxygen-containing adsorbates that assist in the oxidation of poisoning intermediates, whereas Cu lowers the Pt d-band center and facilitates improved adsorption/desorption behavior. Another key finding was that Cu significantly aided gelation acceleration and stabilization of the interconnected aerogel backbone formation, according to the study. Subsequent work shifted attention from activity alone to the simultaneous improvement of activity and durability. Fang et al. introduced trace Ir into PtCu aerogels and proposed that Ir acts as an “adhesive” within the alloy framework [118]. The optimized Pt_1_Cu_1_Ir_0.04_ catalyst showed methanol oxidation mass activity and specific activity that were 4.1 and 2.6 times those of Pt/C, respectively, while retaining 71.2% of its initial mass activity after 20,000 s of chronoamperometric testing. Mechanistic analysis suggested that Ir strengthens metallic bonding within the PtCuIr framework, thereby suppressing Cu dissolution and improving structural robustness (Figure 11a–p).

In parallel, density functional theory indicated that trace Ir lowers the adsorption energy of CO-containing species, which helps mitigate poisoning and promotes faster MOR kinetics. This study is particularly important because it demonstrates that careful compositional tuning in ternary aerogels can directly address one of the major practical limitations of Pt-based MOR catalysts, namely insufficient stability under operating conditions. Morphology engineering has also become a central design principle in this area. A key additional study of oxidative self-assembly of Au/Ag/Pt-alloy nanoparticles into freestanding aerogels was reported by Sarkar et al. [119]. These Au/Ag/Pt aerogels were built from ultrasmall alloy nanoparticles and displayed high surface area, mesoporosity, direct nanoparticle connectivity, and improved conductivity. This work reported that the aerogels achieved 2.5 times the MOR mass activity of commercial Pt/C and retained about 94% of their initial MOR activity after 24 h at constant potential in alkaline medium. The authors attributed the enhancement to the porous interconnected architecture together with the beneficial role of oxophilic Au, which promoted methanol activation and improved tolerance to carbonaceous byproducts.

Li et al. [120] developed PtNi nanotubular aerogels through a dual nanoengineering strategy involving fast co-reduction followed by post-treatment with acid and galvanic replacement (Figure 11q–t). The resulting hollow, interconnected PtNi framework displayed a MOR mass activity of 3.2 A mgPt^−1^ and a specific activity of 5.5 mA cmPt^−2^, corresponding to about 2.1 and 2.6 times the values of Pt/C (Figure 11u–w). Importantly, the same strategy was extended to other multi-component systems, including RuNi, Au–PtNi, and Ru–PtNi aerogels, indicating that nanotubular morphology control can serve as a general route for constructing efficient fuel cell electrocatalysts. This work highlighted that catalytic performance is not governed solely by alloy composition; rather, structural parameters such as hollow architecture, wall thickness, and pore accessibility also exert a major influence on active-site utilization and mass transport. A distinct synthetic route was reported by Sarkar et al., who assembled ultrasmall alloy nanoparticles into self-supported Ag/Pt/Pd aerogels [121]. These aerogels possessed high surface areas of 119.1–140.1 m^2^ g^−1^ and mesopore sizes of 19.7–23.0 nm. The best-performing composition, Ag_0.449_Pt_0.480_Pd_0.071_, achieved a MOR mass activity of 3179.5 mA mg^−1^, approximately 4–5 times higher than commercial Pt/C and Pd/C catalysts, and maintained about 75–90% of its initial mass activity during 17 h chronoamperometric operation in alkaline medium. The authors attributed this behavior to several combined effects, including trimetallic alloying, ligand removal, mesoporosity, and Ag dealloying-induced surface enrichment of Pt and Pd sites. By demonstrating that freestanding trimetallic aerogels derived from nanoparticles can offer both high activity and strong poisoning resistance, this study expanded the conceptual framework of MOR aerogels.

Recent studies have further emphasized the value of fine electronic regulation by noble metal doping. In 2025, Ma et al. reported an Au-doped Pt–Co aerogel, in which the optimized Au1-Pt_67_Co_33_ composition exhibited a mass activity 1.7 times that of the undoped Pt_67_Co_33_ aerogel and 4.6 times that of commercial Pt/C [122]. The catalyst also showed markedly improved long-term stability. Density functional theory calculations suggested that Au doping lowers the energy barrier for methanol dehydrogenation and decreases the overall barrier for CO oxidation, thereby accelerating both fuel activation and intermediate removal. This work illustrates how a small amount of a third metallic component can substantially alter the electronic structure of Pt-based aerogels and improve both catalytic efficiency and durability.

**Figure 12:**
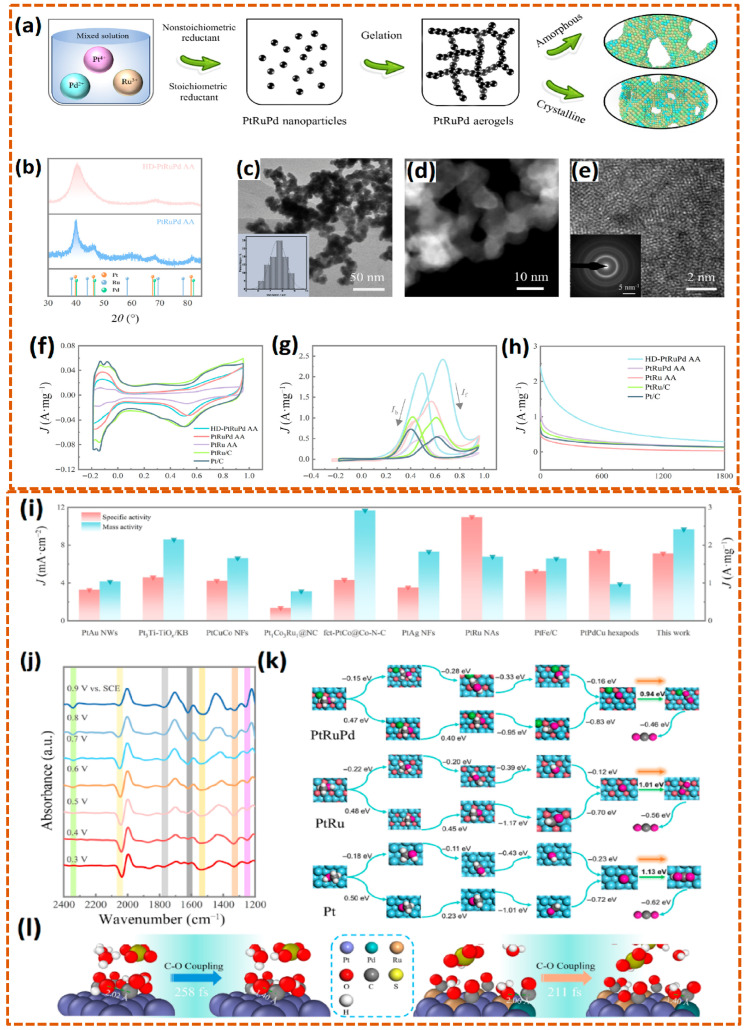
(**a**) Schematic representation of HD-PtRuPd AA, (**b**) XRD diffraction patterns of PtRuPd-AA and HD-PtRuPd-AA, (**c**–**e**) TEM images of HD-PtRuPd AA. CV curves of various HD-PtRuPd AA catalysts along with Pt/C and Pt-Ru/C catalysts in (**f**) 0.5 M H_2_SO_4_ and (**g**) 0.5 M H_2_SO_4_ + 1 M CH_3_OH. (**h**) i-t curves at 0.6 V for 1800 s, (**i**) in situ FTIR spectra under MOR operation for HD-PtRuPd AA, (**j**) Free energy step diagram for Pt, PtRu, and PtRuPd, (**k**) AIMD for MOR in acidic solvent, (**l**) ab initio molecular dynamics for MOR in acidic solvents [obtained from ref. [123], open access].

Guan et al. [123] combined machine learning based on 616 experimental data points with first-principles calculations to identify promising MOR catalyst compositions and subsequently synthesized a highly disordered PtRuPd alloy aerogel. The resulting catalyst exhibited a mass activity of 2.42 A mgPt^−1^ and a specific activity of 7.13 mA cm^−2^ for MOR. More importantly, when integrated as a self-supported ultrathin anode catalyst layer in a membrane electrode assembly, it delivered a mass-specific power density of 92.9 W gPt^−1^ at 65 °C in a direct methanol fuel cell (Figure 12). Not only does this study show that digital screening tools can lead to the discovery of high-performance ternary aerogels with strong half-cell activity and meaningful device-level performance, but it also goes beyond conventional empirical alloy optimization.

**Figure 13:**
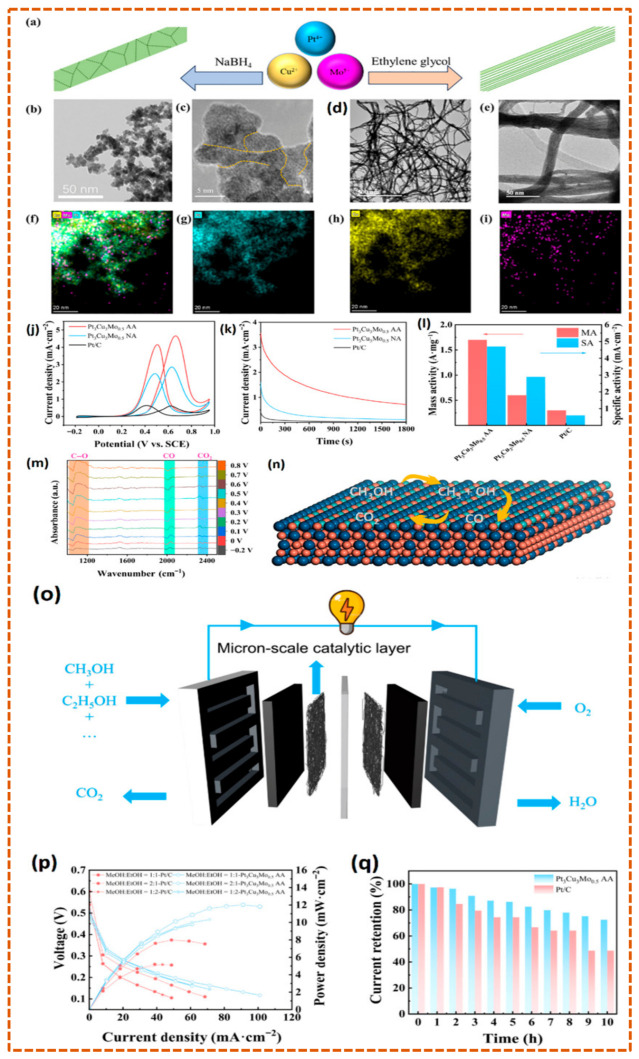
(**a**) schematic presentation of PtCuMO AAs and PtCuMO Nas, (**b**–**e**) TEM images of Pt3Cu3Mo0.5 NA (the yellow boundary lines indicate the phase boundary). The Pt (111) crystal plane and the yellow line segment serve as the grain boundary region. (**f**–**i**) TEM elemental mapping for Pt, Cu, and Mo, (**j**) MOR CV curves, (**k**) I vs. t curves, (**l**) mass and specific activities of various PtCuMO AAs and PtCuMO Nas, (**m**) in situ IR test curves of MOR catalysts, (**n**) Pt3Cu3Mo0.5 AA methanol oxidation reaction pathway diagram. (**o**) Schematic diagram of broad-spectrum direct liquid fuel cells, (**p**) steady state polarization and power density curves of Pt3Cu3Mo0.5 AA and Pt/C anode mixed fuel at 65 °C. (**q**) Stability test of 0.3 V under the condition of 1:1 anode mixed fuel [obtained from Ref. [124], open access].

Defect engineering has also recently emerged as a powerful strategy for tuning MOR performance. Liu et al. [124] reported grain-boundary-engineered PtCuMo alloy aerogels designed for broad-spectrum alcohol oxidation in direct liquid fuel cells. Their optimized Pt_3_Cu_3_Mo_0.5_ aerogel exhibited a 150% enhancement in MOR activity relative to the corresponding nanowire-array comparator and reached an area-specific activity of 4.7 mA cm^−2^, markedly higher than Pt/C. The same material also achieved a mass-specific power density of 14.14 W gPt^−1^ in a mixed methanol/ethanol fuel configuration. Density functional theory analysis indicated that grain boundaries broaden the adsorption energy window of CO* and OH* intermediates, thereby creating a wider distribution of catalytically competent sites (Figure 13).

Overall, the available literature reveals a clear evolution in the design logic of aerogel electrocatalysts for MOR. Early work established that ternary aerogels such as PtRuCu can outperform Pt/C through the combined benefits of porous architecture and alloy synergy. Later studies showed that durability can be strengthened through trace-element incorporation, as in PtCuIr and Au-doped Pt–Co systems. Parallel efforts demonstrated that hierarchical nanotubular structures, dealloyed trimetallic surfaces, highly disordered atomic arrangements, and grain-boundary-rich frameworks can each provide additional gains in activity and resistance to poisoning. Taken together, these reports indicate that the most effective MOR aerogels arise from the simultaneous optimization of composition, electronic structure, morphology, and defect chemistry. Accordingly, ternary and multi-metallic aerogels now represent one of the most versatile and promising platforms for the development of next-generation anode catalysts for direct methanol fuel cells.

### 5.4. High and Medium Entropy Alloy Aerogels

A relatively new concept of high entropy alloys (HEAAs) has recently been gaining tremendous interest in the catalysis research area due to their unique mechanical, physical, and chemical properties, such as high lattice distortion and slow diffusion of metallic atoms [125]. The high entropy alloys are defined by the entropy of larger than 1.5R (R = gas constant), whereas medium entropy alloys (MEAAs) have the R between 1–1.5 [126]. Materials with high entropy can exhibit extraordinary physicochemical, mechanical, and catalytic properties [127]. HEAs are generally composed of five or more metallic elements, giving rise to a vast diversity of metallic composition, leading to hundreds of compositions [128]. In addition, the HEAs exhibit a cocktail effect in which the multi-metallic composition provides multiple sites of adoption to the reactants and intermediates, which is attributed to the significant atomic-level distortions and short-range ordering [129]. Taking advantage of the above facts, several HEAs have been identified as electrocatalysts for a variety of chemical and electrochemical reactions [130]. Ju et al. [131] depicted the synthesis of HEAAs made of five metallic elements, PtBi_1.5_Ni_0.2_Co_0.2_Cu_0.2_, as a MOR catalyst via a simple two-step process. Typically, an aqueous mixture of various metallic precursors was reduced by NaBH_4_ in the presence of β-cyclodextrin as the surfactant and a capping agent. SEM and TEM analysis show that the HEAAs catalyst shows a porous nature and high crystallinity. XRD analysis shows that the sharp peaks are associated with the high crystallinity and the peak positions shift to higher 2 theta values compared to Pt, suggesting the formation of lattice distortions produced by the presence of different metals. Benefiting from the high porosity and the presence of multi-metallic atoms and lattice distortions, the HEAAs exhibited 12.7 times higher mass activities compared to Pt/C in 0.1 M H_2_SO_4_ + 1 M methanol solution. The enhanced MOR performance is attributed to the electron transfer from Ni, Co, and Cu to Pt. The d-band center of PtBi_1.5_Ni_0.2_Co_0.2_Cu_0.2_ alloy was found to be −3.957 eV, lower than the PtBi_1.5_ and Pt/C, which are around −3.795 and −3.705 eV. The lower shift of the d-band center contributes to the weaker *CO adsorption, leading to enhanced MOR activity. When applied as a cathode catalyst in single-cell DMFCs, a maximum power density of 115 mW cm^−2^ was achieved, which is 1.5 times higher than that of the Pt/C catalyst. Therefore, the HEAAs not only exhibited good MOR activity in three-electrode configurations, but also in practical single-cell DMFCs, showing promising applications as cathodes in methanol fuel cells.

In another study, Lu et al. [132] synthesized PtPdRuCuNi alloy aerogels featuring a self-supporting network structure as high-efficiency catalysts for the MOR. In typical synthesis, equimolar concentrations of metallic aqueous precursors were reduced by the NaBH_4_ reduction method. The excess NaBH_4_ not only works as a reducing agent but also acts as a salting-out agent to accelerate the metal nanoparticle aggregation and fusion of the nanoparticles. The rapid evolution of H_2_ gas collides with metallic atoms, promoting the attachment and the gelation process. The morphological observation of the metal aerogels was found to have a nanowire structure with a ligament size of 5.16 nm. The electrochemical measurements indicate that PtPdRuCuNi alloy aerogels exhibit a lower potential for methanol oxidation, suggesting enhanced methanol oxidation kinetics, which is attributed to the electron transfer among the alloying elements synergistically. The PtPdRuCuNi alloy aerogel exhibited mass activities higher than the Pt-Ru/C catalyst, which are 2.05, 1.37, and 4.50 A mg_Pt_^−1^ in acidic, neutral, and alkaline electrolytes, specifically exhibiting the highest MOR activity in alkaline electrolytes. When applied as a cathode catalyst in single-cell DMFCs, a maximum power density of 77.7 mW cm^−2^ was achieved, which was slightly higher than that of the Pt/C and Pt-Ru/C catalysts. In another study, Li et al. [133] demonstrated the effect of Pd, Au, Cu, Co, and Mo on the PtNi catalysts by synthesizing a variety of high entropy alloy materials. The obtained Pt_40_Ni_30_M_30_ (M = Co, Pd, Au, Mo, Cu) aerogels exhibited enhanced MOR activity towards alkaline MOR, following the activity trend, Pt_40_Ni_30_Pd_30_ > Pt_40_Ni_30_Au_30_ > Pt_40_Ni_30_Cu_30_ > Pt_40_Ni_30_Co_30_ > Pt_40_Ni_30_Mo_30_, with mass activity improvements of 12.7, 5.9, 5.4, 4.4, and 4.3 times higher than that of Pt/C, respectively. These results demonstrate that HEAAs could be applied as MOR catalysts in methanol fuel cells.

It is well known that Pt has a high adsorption capacity for *Co intermediates that results in catalyst deactivation. Like Ru’s role as a waste dissociation agent that provides the *OH, another element, Cu, also catalyzes the water dissociation. On the other hand, Pd is known to modify the Pt electronic structure and d-band center engineering when they are alloyed, which enhances the electron movement and helps rapid electron transfer between them. Based on the electronic configuration of coupling d orbitals of Pt-5d/Pd-4d/Cu-3d, it is possible to optimize the d-band center of Pt to achieve high-performance DMFCs. Based on this logic, Wang et al. [134] synthesized PtPdCu MEAAs via a simple surfactant-free, one-step, sodium borohydride method. The strong electronic interaction between metals was seen by the positive shift in their peaks relative to their metallic state of Pt and Pd, whereas the Cu peaks shifted negatively, which can be attributed to the charge transfer between Pt, Pd, and Cu atoms. The synthesized PtPdCu MEAAs exhibited relatively higher electrocatalytic activity for MOR in acidic, alkaline, and neutral pH electrolytes, compared to Pt/C and the monometallic aerogels. Interestingly, the PtPdCu MEAAs also exhibited higher power density in DMFCs with an ultra-low catalyst layer thickness of 4–5 µm with a catalyst loading of 2 mg/cm^2^, and a control sample of Pt/C with the same catalyst loading of 2 mg/cm^2^, which leads to a catalyst layer thickness of ~40 µm. The obtained power density was 35 and 20 mW/cm^2^, respectively, for PtPdCu MEAAs and the Pt/C catalyst. This study clearly distinguishes the advantage of metal aerogels in terms of significantly reducing the catalyst layer thickness by several times, reducing resistance related to mass transport issues. In addition, an impressive stability of the PtPdCu MEAAs electrode was also noticed at a constant current of 50 mA/cm^2^ for 10 h, and the resulting voltage decreased by 24%. These results suggest that the d-d orbital coupling could be one of the strategies to optimize the metal aerogel catalyst MOR activity.

### 5.5. Non-Pt Metal Aerogels

Due to the high cost of Pt and noble metal-based catalysts, the development of non-Pt-based catalysts has become paramount to reducing the cost associated with the catalysts of DMFCs [135]. Specifically, non-Pt-based metal aerogel catalysts are rarely investigated. Therefore, there is an urgency to develop cost-effective, non-Pt-based metal aerogels with high MOR activity for MOR. Among several transition metal-based metal aerogels, Fe and Ni-based electrocatalysts have gained interest due to their strong electronic interaction that would optimize the d-band center for the optimal adsorption of reactants and intermediates to enhance faster electron transfer and improved electrocatalytic activity [136,137]. As a result of the insufficient dispersion of nanosized alloys on carbon supports and inadequate exposure of active sites, the electrochemical activity of most catalysts based on iron and nickel is low. In addition, the corrosion of the carbon support further decreases the stability of electrocatalysts. The first non-Pt-based metal aerogel was reported in 2015 by Zhu et al. [138]. A novel hollow nickel-cobalt-oxide-based nano sponge morphological aerogel was synthesized by a NaBH_4_ strategy. The 3D bimetallic nano sponge metal oxide (Ni-Co oxide HNSs) was obtained by the annealing of the metal aerogel at 600 °C. The obtained Ni-Co oxide HNSs were then applied as an OER electrocatalyst. Following this report, Dubale et al. [139] synthesized oxophilic post-transition metal Bi-doped Ni aerogel as a MOR electrocatalyst. The as-obtained Ni_97_Bi_3_ aerogel electrocatalyst exhibited exceptional electrocatalytic activity and stability in MOR. It was found that a trace amount of Bi on Ni aerogel resulted in significant compressive strain and the optimum shift on the d-band center of Ni that improves the electronic conductivity of the Ni_97_Bi_3_ aerogel catalyst. The morphological observation by SEM and TEM of the Ni_97_Bi_3_ aerogel catalyst shows the 3D porous structure comprising a network of nanowires, which are interconnected randomly. Interestingly, the control sample made of Bi aerogel shows a flake-like morphology, whereas Ni aerogel and Ni_97_Bi_3_ aerogel show nanowire morphology. The TEM elemental mapping confirms the presence of thicker nanowires and the presence of Ni, Bi, and O. The overall element distributions display a core-shell-like character with Ni, Bi, and O in the shell and Ni primarily in the core. The formation of core-shell structures might be associated with the oxidation of the Ni-Bi nanoparticles during exposure to air. The XPS results suggest that the Ni on the surface of Ni_97_Bi_3_ is mainly metallic, but on the surface, it is mainly in an oxidized state. As for the electrocatalytic activity in methanol oxidation of the as-prepared aerogels, the Ni_97_Bi_3_ aerogel revealed the highest MOR activity in terms of mass current density. In a recent study by Zhang et al. [140], a synthesized core-shell structured Fe@Ni aerogel proved to be an efficient bifunctional electrocatalyst for both OER and MOR. The FeNi_2_ aerogel was prepared by the traditional sol-gel method. The morphological analysis revealed a unique 3D porous structure with FeNi_2_ nanoparticles that interconnected with each other, and the EDS showed the Fe@Ni core-shell structures. When applied as a MOR catalyst in 1 M KOH + 1 M CH_3_OH electrolyte, the FeNi_2_ aerogel exhibited a specific activity of 213.6 mA cm^−2^ and a mass activity of 106.8 A g^−1,^ which are notably higher than the control samples of Ni and Fe aerogels. To the best of our knowledge, the literature studies on non-noble metal aerogels are very limited. This indicates that there is tremendous room for the development of metal aerogels made of non-precious metals for MOR.

**Figure 14:**
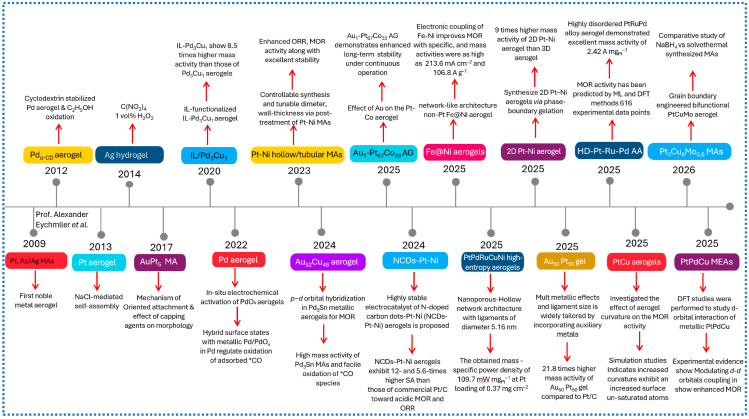
Simplified picture of important studies on metal aerogel catalysts from the years 2009 to 2026 [Pt, Au/Ag MAs [82], Pd_α_-CD aerogel [141], Pt aerogel [142], Ag hydrogel [143], AuPt_5_ MA [81], IL/Pd_3_Cu_1_ [103], Pd aerogel [106], Pt-Ni hollow/tubular MAs [120], Au_52_Cu_48_ aerogel [116], Au_1_-Pt_67_Co_33_ AG [122], NCDs-Pt-Ni [98], Fe@Ni aerogels [140], PtPdRuCuNi high-entropy aerogels [132], 2D Pt-Ni aerogel [94], Au_50_ Pt_50_ gel [144], HD-Pt-Ru-Pd AA [123], PtCu aerogels [145], Pt_3_Cu_6_Mo_0.5_ Mas [124], PtPdCu MEAs [134]].

To summarize, metal aerogel-derived electrocatalysts have demonstrated exceptional MOR activity, with mass activities that are two to 22 orders of magnitude higher than those of commercially available Pt/C and Pt-Ru/C metal catalysts in a traditional three-electrode system. Since their discovery in 2009, significant progress has been made in terms of synthesis strategies, mechanisms for destabilization, enhancement of activity, and efforts to understand the effect of metal aerogel thickness and stability in electrochemical conditions with regard to these materials. With regard to the metal aerogel electrocatalysts for MOR, the significant milestones are depicted in Figure 14. Since their discovery in 2009, specifically focusing on the advancements of MOR catalysis [81,82,94,98,103,106,116,120,122,124,132,140,141,142,143,144,145,146,147], it can be concluded that metal aerogels possess great potential as efficient electrocatalysts for MOR, provided their potential in a realistic fuel cell is established. Table 2 displays the MOR kinetics data of various metal aerogel catalysts in comparison with the standard catalysts. Several metal aerogel-based catalysts exhibited enhanced MOR performance when compared to their control catalysts or the standard, commercial catalysts. However, it should be noted that a direct comparison of the MOR performance of various metal aerogel catalysts would be difficult due to variations in the catalyst composition, physicochemical properties, and evaluation conditions such as the composition of electrolyte and testing methods, etc. Readers are encouraged to compare their results more cautiously based on various factors, such as catalyst composition, physicochemical properties, and the evaluation conditions of metal aerogels based on the Table 2 compilation data. The data shown in Table 2 must be interpreted based on the context of the specific catalyst evaluation method and catalyst composition. In the present format, Table 2 and Table 3 signify the potential of the metal aerogel catalyst over the standard and control samples that are assumed to be tested at similar testing conditions. The inconsistency of the testing conditions calls for the prioritization of standard testing protocols for easier comparison.

## 6. MOR Performance Comparison of Metal Aerogels with Other Catalyst Systems

**Figure 15:**
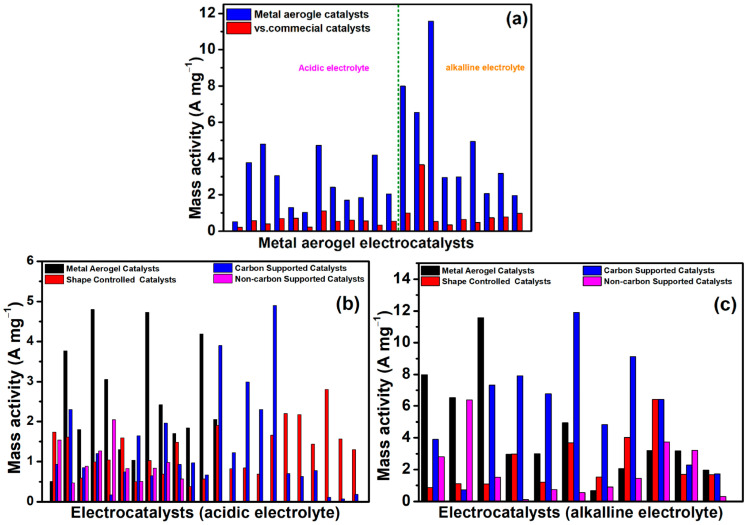
Comparison of mass activity of metal aerogel catalysts with (**a**) commercial catalysts, (**b**) shape-controlled, (**c**) carbon-supported, non-carbon-supported catalysts. The graphs are drawn from Table 1, Table 2 and Table 3, showing mass activity values, respectively.

Upon reviewing the literature, we discovered that metal aerogel-based catalysts demonstrated superior performance relative to state-of-the-art commercial catalysts, including Pt/C and Pt-Ru/C catalysts. Figure 15 illustrates the comparative mass activities of metal aerogel catalysts in relation to standard catalysts. It is important to note that, although standard catalysts are sourced from commercial suppliers, the mass activity values differ due to variations in catalyst testing protocols, discrepancies in catalyst ink preparation, binder compositions, and differing electrolyte formulations employed by various researchers. To prevent this misinterpretation, we have specified the mass activity of the standard catalyst in each study. This will facilitate a direct comparison of the efficacy of metal aerogel catalysts against standard catalysts under analogous testing protocols. The metal aerogel demonstrated superior performance relative to carbon-supported commercial catalysts, with enhancements ranging from 2-fold to 22-fold. Moreover, the metal aerogel catalysts demonstrated superior mass activity in alkaline methanol oxidation reactions, subsequently followed by acidic methanol oxidation reactions. Additionally, we have evaluated the efficacy of metal aerogel electrocatalysts against various advanced shape-controlled electrocatalysts, carbon-supported electrocatalysts, and non-carbon-supported electrocatalysts in both acidic and alkaline electrolytes [148,149,150,151,152,153,154,155,156,157,158,159,160,161,162,163,164,165,166,167,168,169,170,171,172,173,174,175,176,177,178,179,180,181,182,183,184,185,186,187,188,189,190,191,192,193,194,195,196,197,198,199,200,201,202,203,204,205,206,207,208,209,210,211,212,213,214,215,216,217,218,219,220,221,222,223,224,225,226,227,228,229,230], as shown in Table 3 and Figure 15. In acidic electrolytes in particular, metal aerogel catalysts outperform other catalytic systems in terms of mass activity, which is an interesting finding. It is evident from these statistical analyses that metal aerogel catalysts have the potential to be used as anodes in DMFCs.

## 7. Critical Analysis, Structure-Activity Relationship, and Cross-Comparison of Various Metal Aerogel Catalysts

After reviewing all the possible literature of metal aerogel catalysts for MOR activity, one common thing that was observed is the synthesis of metal aerogels from the aqueous solutions of metal salts from NaBH_4_ solution. Some of the studies utilized stabilizing, capping agents [81]. For example, dopamine, citric acid, and beta-cyclodextrin, etc., have been proposed as a structural directing agent for oriented attachment of nanowires [93]. However, it is not clear whether the capping agents have been removed from the catalyst surface or not, or how they affect the MOR activity. Another common aspect that we observed is the morphology of the catalysts, while most of the metal aerogel catalysts exhibit highly crystalline morphology with interlinked network structures composed of ultrathin nanowires (Table 2), some studies also reported dendritic structures [231]. In addition, bimetallic and trimetallic metal aerogels exhibit alloying effects, which is also one of the important reasons for the enhanced MOR activity. In terms of the BET surface area, except for [116,119,121], almost all of the catalysts exhibited a surface area of an average of ~50 m^2^ g^−1^, which is lower than the traditional carbon-supported catalysts. The low surface area of metal aerogels arises due to metal nanoparticles being interconnected and interlinked, unlike nanosized metal particles dispersed on the carbon support. The scientific community has been driven to investigate alternative methods and materials due to growing production costs, which are caused by costly metal precursors and drying techniques. In this regard, the synthesis of 2D Pt–Ni aerogel proposed by Alexander Eychmüller et al. [94] via a phase-boundary gelation method is an interesting work, which eliminates the need for special drying methods and uses a small amount of metal precursors. Despite their significant potential as high-efficiency electrocatalysts, metallic aerogels encounter challenges, including metal dissociation, aggregation, and deactivation during prolonged operation, which compromise their electrocatalytic durability. Integrating metallic catalysts with carbon materials is viable for enhancing stability and optimizing activity due to effective anchorage, excellent conductivity, high surface area, and the improved dispersion of metal catalysts within carbon materials. In this regard, a unique strategy proposed by Li et al., in which -doped carbon dots are integrated, is a stand-out strategy [98]. Although integrating carbon-based materials into metal aerogels goes against the fundamental advantage of being carbon-free, we believe confining the carbon into the metal aerogel framework, unlike the traditional carbon support, boosts the activity, especially the stability against the Ostwald ripening and aggregation of the metal aerogels under long operating conditions. Furthermore, confining the carbon-based materials inside the metal aerogel network boosts the electronic conductivity and enhances the interaction between carbon-metal active sites. As is evidenced in the study, the NCDs-PtNi exhibited admirable stability. Among all the metal aerogel catalysts that we investigated in this review, the ligand size modulated the Au_50_Pt_50_ gel, where a ligament size controlling strategy was proposed based on the unique sedimentation-based sol-gel behavior of metals by utilizing the average bulk density (r_ab_) and atomic radius (r_a_) mismatch [144]. Using this strategy, the ligament size was dramatically reduced (up to >90%), and the Au_50_Pt_50_ gel catalyst exhibited 21.8 times higher MOR activity than the Pt/C. This catalyst stands out as the top catalyst in this review article. On the other hand, the bimetallic metal aerogel catalysts exhibited enhanced MOR performance over monometallic aerogels due to the fact that the alloying effect and bifunctional mechanisms offered by secondary metals, along with the alloying effect, downshift the d-band center. Enhanced MOR activities have been seen for metal aerogels of bimetallic and trimetallic catalysts over conventional carbon-supported catalysts, as shown in Table 2. Another interesting work describes the synthesis of three-dimensional porous PtNi aerogels composed of PtNi alloyed nanotubes with tunable diameter and wall thickness; these were designed through a nanoengineering strategy of gelation and post-treatment proposed by Li et al. [120]. This strategy was proposed initially for PtNi aerogels but also extended to other multi-component metallic nanotubular aerogels, such as Ru–PtNi and Au–PtNi aerogels. The morphology of the nanotubular PtNi proposed in this study is considerably different from that of the metal aerogels of nanochain-based metal aerogels. However, the mass activities of the nanotubular structures were found to be very similar to the nanochain type of metal aerogels.

From the cross-comparison of various metal aerogels, Pd_3_Sn MAs synthesized via ionic liquid, as the initiator, and N_2_H_4_, as the reductant, exhibited the highest BET surface area of 190 m^2^ g^−1^, exhibiting a 3D highly porous structure beneficial for enhancing the mass transport properties during MOR reaction and facile CO_2_ removal [116]. A medium surface area of 125–140 m^2^ g^−1^ was reported for the Ag/Pt/Pd and Au/Ag/Pd catalysts [119,121]. Most binary metals of Pt and HEA-based catalysts showed a moderate to high BET surface area of ~20–60 m^2^ g^−1^. From the ECSA point of view, Cu alloyed Pt and Pd catalysts [84,103] have shown the highest ECSA values, followed by Pt-Bi and NCDs-PtNi catalysts [98,146]. In addition, the Pt-Bi catalyst showed the highest mass activity of 7.99 A mg^−1^ among Pt-based binary catalysts. In terms of MOR activity in alkaline electrolytes, Au_50_ Pt_50_ gel > Pt-Bi > PtRu catalyst showed excellent MOR activity [144,146,147], whereas in acidic electrolytes, NCDs-PtNi > Pt_1_Cu_1_Ir_0.04_ catalyst exhibited excellent MOR activity [98,118]. In cross-analysis of MOR over benchmark catalyst, NCDs-PtNi exhibited 12 times higher MOR activity in acidic electrolytes, whereas Au_50_Pt_50_ gel catalysts exhibited 21.8 times higher MOR activity than the Pt/C. However, readers should note that these comparisons were merely based on the mass activities; authors are advised to refer to Table 2 and Table 3 for detailed half-cell evaluation conditions, catalyst composition, and catalyst morphology, and the structure–activity relationship from the original works, for fair comparison.

## 8. Activity Enhancement Factors of Metal Aerogel Catalysts

**Figure 16:**
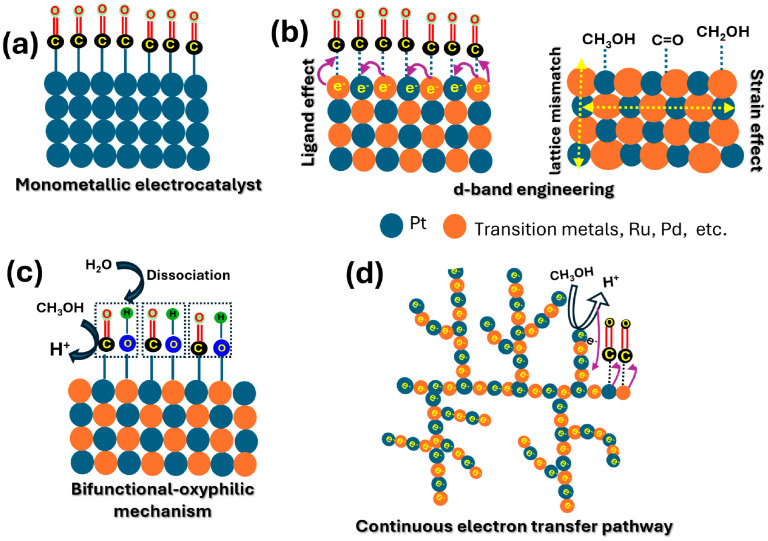
MOR activity enhancement factors (**a**) strong adsorption of CO species, (**b**) d-band engineering by ligand and strain effects, (**c**) bifunctional oxyphilic mechanism [56], open access], and (**d**) continuous electron transfer pathway in metallic aerogels.

The enhanced MOR activity of metal aerogel electrocatalysts comes from the self-supporting nanochain morphology with 3D porous architecture and a completely metallic backbone that guarantees the uninterrupted, facile electron movement contributing to the enhanced electronic conductivity during MOR. Apart from the morphological benefit, the intrinsic catalytic performance of a particular catalytic surface is the source of the metal aerogel electrocatalysts’ increased mass and specific activities, which are essentially dictated by the electronic structure and coordination environment of the surficial active sites. (i) adjusting the d-band center, and (ii) the bifunctional oxyphilic effect mechanisms, as explained below, are two significant factors that contribute to increased MOR activity (Figure 16).

**Figure 17:**
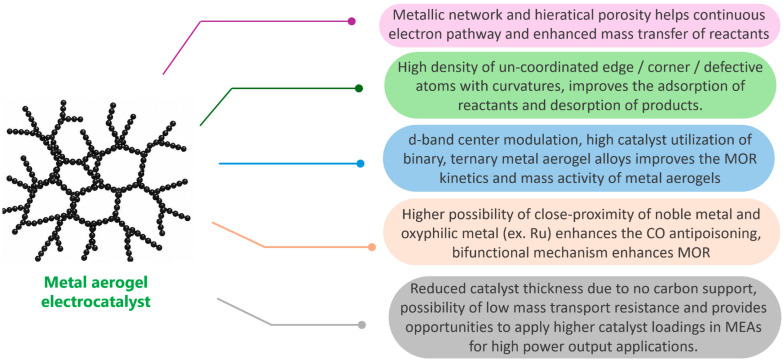
Summary of the important properties of metal aerogels that are beneficial towards MOR in DMFCs.

(i) d-band center tuning: The d-band center of the catalysts determines the adsorption energy strength between the metallic catalyst surface and reactants and the reaction intermediates [232]. An optimum catalyst must contain a balanced adsorption strength that is neither too strong nor too weak. In general, a downshift of the d-band center relative to the Fermi level is usually associated with the weakened (towards optimum) adsorption energy during MOR. The d-band center regulation is a popular concept in electrocatalysis, which will be tuned either by ligand effect or strain effect [233]. The ligand effect is established by combining two or more metallic constituents into an alloy catalyst. It is believed that the coordination environment of the metallic sites, the composition, and the electronic interaction between the alloying elements significantly affect the position of the d-band center [234]. The d-band center of an electrocatalyst can also be fine-tuned by the strain effect [235]. The strain effect arises due to lattice mismatch between the different metallic atoms, which is believed to be a responsible factor for enhanced MOR activity. The strain effect also greatly facilitates surface -OH formation on oxyphilic elements (ex, Ru) adjacent to the noble metal, such as Pt, to help remove CO. Among almost all the metal aerogels that are bimetallic, trimetallic, and multi-metallic catalysts, MOR activity has been attributed to the ligand and strain effects.
(ii) Bifunctional mechanism: The formation of CO on the noble metal surface is an important limiting factor for MOR catalysis. The strong adsorption of CO on the Pt surface leads to decreased catalyst surface area available for the MOR reaction, leading to CO poisoning of the catalysts. It is well documented that the presence of oxyphilic elements adjacent to noble metals helps the removal of CO from the Pt surface by H_2_O oxidation at lower potentials that provide -OH groups, helping to combine CO + OH into CO_2_ [56,236]. Metal aerogels of bi-, tri-, and multi-metallic catalyst MOR activity have been attributed to the bifunctional mechanism.
(iii) Defects, coordination, environmental, and curvature effects: At the atomic level, the strain effect brought on by the curvature of the near-surface region coexists with the geometric effect caused by high-density low-coordination atomic sites at the edges, steps, and kinks, maximizing the catalyst’s catalytic activity and number of active sites [237,238,239]. The low curvature makes the catalyst more stable and resistant to Ostwald ripening [240]. There is a strong correlation between the surface coordination number and the electronic structure (e.g., the position of the d-band center), and the adsorption behavior (e.g., reaction rate, selectivity, and stability) of active sites on catalyst surfaces, which in turn greatly affects catalytic activity [241]. For low-coordination numbers (e.g., corner/edge atoms), increased unsaturated bonds in atoms lead to more localized d-electrons, serving as primary sites for the activation of reactants. In this regard, the metal aerogels with hierarchical nanochain morphology and curvature allow the high density of unsaturated coordination bond sand defects to greatly influence the MOR activity. This concept has been proven by Liu et al. [145] in PtCu-HC aerogel catalyst performance. The PtCu-HC aerogel (obtained at 45 °C) exhibits refined ligaments, increased branching, and higher curvature, generating abundant high-surface-energy active sites and enhanced MOR activity. In conclusion, the metal aerogels exhibit several interesting properties that are beneficial towards MOR in DMFCs (Figure 17).

## 9. DMFC Fuel Cell Performance of the Metal Aerogel Catalysts

Although most of the metal aerogel catalysts have been evaluated in half-cell studies, very few studies have actually analyzed the practical ability of metal aerogels in DMFC single cells (Table 4). For example, Wang et al. [103] employed an IL/Pd_3_Cu_1_ catalyst in DMFCs. The DMFC single-cell with IL/ Pd_3_Cu_1_ as anode catalyst developed an OCV of 0.54 V and a power density of just 8 mW cm^−2^; the obtained power density was higher than Pd aerogel and Pd/C catalysts by 1.7 and 7 times. In another study, Guan et al. [123] applied HD-PtRuPd aerogel as anode in DMC and obtained a power density of 60.4 mW cm^−2^, which is higher than the counterpart catalysts PtRu/C and Pt/C, which delivered a power density of 36.3 and 31.7 mW cm^−2^. In another study, Liu et al. [124] applied Pt_3_Cu_3_Mo_0.5_ aerogel as anode catalyst with a catalyst loading of 0.85 mg_Pt_ cm^−2^, in a mixture of methanol + ethanol in the ratios of 1:1, 1:2, and 2:1, obtaining power densities of 12.02, 10.33, and 9.75 mW·cm^−2^, respectively, and for Pt/C as anode, the power densities were 7.99, 5.20, and 5.16 mW·cm^−2^, respectively. In a very recent study. Wang et al. [134] employed PtPdCu MEAAs erogel catalyst in DMFCs with a PtPd loading of 2 mg cm^−2^; the maximum power density of 35 mW/cm^−2^ was achieved in acidic electrolytes, which is 1.8-fold more than that of the commercial Pt/C with 20 mW/cm^2^. In another study, Ju et al. employed high entropy alloy catalyst PtBi_1.5_Ni_0.2_Co_0.2_Cu_0.2_ in DMFCs, which delivered a power density of 115 mW cm^−2,^ which is 1.5 times higher than that of Pt/C with the same Pt loading. In another study, Lu et al. [132] employed PtBi_1.5_Ni_0.2_Co_0.2_Cu_0.2_ HEAAs high-entropy alloy aerogels in DMFCs, and the alloy aerogel delivered a power density of 77.7 mW cm^−2^ at 65 °C, and the mass-specific power attained a remarkable 109.7 mW mgPt^−1^ at Pt loading of 0.37 mg cm^−2^.

Table 4 shows the fraction of studies that reported the single-cell DMFC performance of metal aerogel catalysts, while most of the metal aerogel catalysts have only been evaluated in the half-cell configuration. Although the fundamental electrochemical analysis of metal aerogel catalysts in a traditional three-electrode system is significant to gain knowledge on intrinsic electrocatalytic activity and mechanistic studies, for practical application, it is important to measure the power density analysis in a more realistic condition. This is an important requirement due to the fact that the mass activity values that are obtained in half-cell studies do not directly translate into the realistic DMFC configuration.

## 10. Challenges in Half-Cell Studies vs. Realistic DMFC Studies

In general, the half-cell studies are designed to isolate the electrocatalytic activity of the catalyst under idealistic conditions, such as very thin catalyst loading, a large quantity of the electrolyte ensures the effective three-phase contact, with no cathode catalyst, and a membrane completely isolates the anode catalyst performance from other influencing factors. However, for realistic DMFCs, several factors dominate the overall performance as discussed below.

First, the fundamental difference between half-cell and realistic DMFCs arises from the electrode architecture and the mass transport-related resistances. In half-cell studies, the electrode catalyst layer is very thin and is in contact with a large amount of liquid; therefore, the resistance arising from the mass transport of CH_3_OH reaching the electrode surface is less in half-cell studies, whereas in DMFCs, the electrode architecture is made of a carbon backing layer, gas diffusion layer, and catalyst layer. Therefore, the mass transport of CH_3_OH to the electrode surface is associated with the diffusion-related resistances. In addition, half-cell studies employ very low amounts of catalyst loading (generally in µg), whereas in DMFCs, the catalyst is generally loaded in milligram levels. This significantly increases the catalyst layer thickness and the amount of methanol oxidation current that is produced, and hence the CO_2_ formation that needs to be moved out through the porous structures. In this regard, metal aerogels may be interesting due to the fact that they do not contain any carbon; therefore, the catalyst loading is reduced to a greater extent compared to the traditional carbon-supported catalysts, thus reducing the catalyst layer thickness and related resistance. In addition, the metal aerogels also possess hierarchical porosity, which helps the CH_3_OH diffusion to the catalyst layer and also the facile CO_2_ removal_._ However, it is not clear or guaranteed that the metal aerogel hierarchical porosity remains on MEA in the same way it does in the freshly prepared condition.

Second, the mass activities are measured in half-cell studies at a fixed potential against the idealistic reference electrode. However, in a realistic fuel cell, the over-potential arises from the anode and cathode, and a solid electrolyte matters a lot. Therefore, the DMFC performance will be affected by cell voltage, methanol crossover, electronic conductivity, and mass transport issues.

Third, the stability tests are normally conducted from a few minutes to a few hours in half-cell modes, whereas in realistic DMFCs, the stability needs to be conducted for longer periods to assess the catalyst performance at realistic conditions, such as high concentration of methanol solution and higher temperatures, depending upon the type of DMFC application.

Fourth, ionomer distribution could be one of the important aspects for effectively delivering the power density, which depends on three-phase contact. In half-cell studies, the catalyst layer is in contact with a liquid electrolyte, whereas in DMFC studies, the solid-polymer electrolyte is in contact with the catalyst layer. Therefore, catalytically active site density in half-cell studies is generally high, whereas in MEA configuration, it is relatively lower. This is generally the reason the ECSA from half-cell studies and MEA studies differ substantially.

Fifth, the mass activity of the metal aerogel catalysts is obtained by normalized current with catalyst loading. In half-cell studies, the catalyst architecture with longer nanochain morphology ensures excellent conducting pathways for the electrons at the lower catalyst loading, whereas in MEAs, the higher catalyst loading and hot-pressing, which is applied when making MEAs, potentially disrupts the 3D connectivity and compresses the porous structure. In addition, the longer nanochain morphologies might undergo mechanical disintegration into the smaller ligaments, losing the conducting pathways during catalyst ink preparation. These directly affect the MOR activity in DMFCs.

Lastly, half-cell studies employ catalyst activity assessment over a much smaller geometrical area on the electrode, producing micro to milli ampere currents, whereas in DMFCs, the often larger geometrical areas are used to deliver higher current, generally in amperes. At such higher currents, thermal management, water management, catalyst degradation, and fuel cross over matters most.

Due to these factors, it is often believed that mass activities of any electrocatalysts assessed through half-cell studies do not directly translate to the MEAs, and it is not always guaranteed. Therefore, it is highly essential to not only evaluate the electrocatalytic activity of metal aerogels in half-cell, but also in realistic DMFCs in MEA configuration for assessing the realistic applications.

## 11. Cost and Large-Scale Manufacturing Feasibility

From the commercialization point of view, processability, scalability, and manufacturing feasibility at larger quantities are important factors. From this point of view, since the first developed metal aerogels only date back to 2009, the research on metal aerogels is still in an early stage of development. Since then, the research progress has been slow, primarily due to the high cost of noble metal precursors, longer gelation times, an insufficient number of gelation agents and their mechanistic understanding, relatively larger catalyst cost from the need for sophisticated supercritical drying techniques that are expensive, and energy-intensive systems. To the best of our knowledge, only a fraction of studies have been devoted to the synthesis of metal aerogels at milligram to gram levels [242,243]. There has been a recent study that reported on the motor-based synthesis of metal aerogels [244], metal aerogel synthesis mediated by salts [57], and thin film-coated metal aerogels by spray coating [245]. Nevertheless, methodical studies devoted to industrial-scale manufacturing processability are highly desirable. Further, to reduce the cost and dependency on noble metals, research on non-noble metals-based metal aerogels is highly needed.

## 12. Future Perspectives and Research Directions

**Figure 18:**
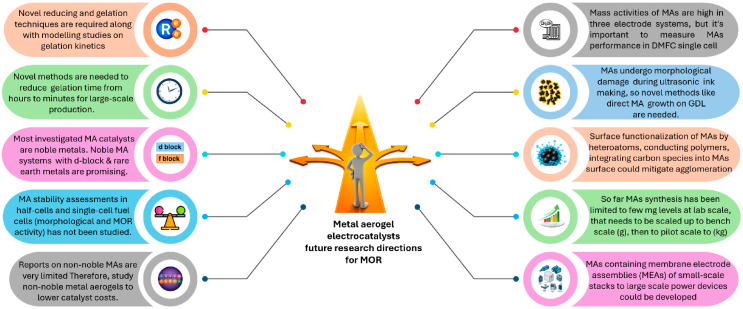
Schematic representation of metal aerogel electrocatalysts and future research directions.

Although the metal aerogel catalysts have shown excellent MOR currents and mass activities, their commercial use in DMFCs is still challenging due to several limitations (Figure 18). We propose several research directions and recommendations for the researchers, as follows.

1. From the perspective of aerogel synthesis, NaBH_4_ has predominantly served as the reducing agent. While the NaBH_4_-based reduction method currently yields satisfactory to excellent electrocatalysts, it is essential to investigate alternative reduction techniques that may alter the morphology, growth kinetics, size, or any fascinating properties of metal aerogels.
2. Another synthesis aspect requiring particular attention is the kinetics, mechanism, and duration of gelation. Since its discovery, researchers have significantly reduced the gelation time from several weeks or days to a few hours; however, the gelation time for most metal aerogels remains approximately between 6 and 24 h. From a commercial perspective, it is crucial to decrease the gelation time from hours to minutes for large-scale production. This advancement is feasible only when the kinetics and mechanism of gelation are thoroughly comprehended to identify the necessary steps that must be adjusted for expedited gelation processes. Understanding both the experimental and modeling aspects of metal aerogel gelation and its kinetics may provide solutions.
3. From the point of MOR activity, it is clearly confirmed that the metal aerogel catalysts possess great potential as an alternative catalyst. However, the MOR activity assessment has only been performed in an ideal three-electrode system. While assessing the electrocatalysts in an ideal three-electrode system serves as a fundamental process, the ultimate applications can only be realized in a real full-cell environment. In addition, the mass activities that are obtained in three-electrode systems often fail to deliver similar results, due to more complex electrode architecture and gas–liquid boundary, three-phase boundary, and CO_2_ removal, etc. In our observation, very few to none of the metal aerogel catalysts have been assessed in a single-cell environment. Therefore, realistic power density measurements are badly required for metal aerogel catalysts.
4. Stability of the electrocatalyst is an important aspect, especially in the realistic DMFC conditions. Unfortunately, none of the metal aerogel catalysts have been evaluated for their stability. Only a few studies have performed chronoamperometric studies for relatively very short periods of time (usually 3600 s), which is nowhere near what can be considered the standard for evaluating the stability of the catalyst. Therefore, it is recommended for researchers to assess the stability of the catalyst for longer durations and accelerated stability tests by repeated cyclic voltametric studies and galvanostatic/potentiostatic tests, and assess the electrochemical and morphological stability of the metal aerogels in comparison with the traditional catalysts. In addition, there exists a lot of inconsistency in the stability evaluation methods; some use CV, and some use CA. In both cases, the stabilities have been calculated for only a few minutes and for a few cycles, which are not sufficient in terms of the long-term stability of catalysts that is required for commercial DMFCs. Therefore, it is highly essential to develop standardized stability protocols and longer durability studies.
5. Despite their significant potential as high-efficiency electrocatalysts, metallic aerogels frequently encounter issues such as metal dissolution, aggregation, and deactivation during prolonged operation, which compromise their electrocatalytic durability. To address the aforementioned challenges, the integration of metallic catalysts with carbon materials is advantageous for enhancing stability and optimizing activity, owing to effective anchorage, superior conductivity, extensive surface area, and the improved dispersion of metal catalysts provided by carbon materials. In this context, incorporating carbon-based conductive materials as a thin coating on the surfaces of metal aerogels may reduce metal dissolution and aggregation. Moreover, investigations into the morphological alterations of metal aerogels following stability tests have been infrequently conducted; thus, it is advisable to perform long-term stability assessments to accurately evaluate the efficacy of metal aerogels as anodes in DMFCs.
6. One crucial aspect of evaluating the electrocatalyst in DMFCs is the preparation of the catalyst ink, which necessitates subjecting the catalyst to an ultrasonic process. The stability of metal aerogels under ultrasonication conditions remains uncertain. It is believed that the inherited metal aerogel structures might be destroyed during the ink preparation. To prevent structural damage, a silicon oil-based method has recently been proposed [246]; however, there is a potential risk of silicon contamination in these instances. Consequently, unique methods for preparing catalyst ink are essential.
7. From the catalyst activity tuning point of view, coupling the metal aerogels with transition metals (oxides)/inner transition metal (oxides) could create the metal-metal oxide that modifies the electronic structure of the catalyst systems that could affect the MOR activity. Further, transition metal oxides have shown excellent co-catalytic activities and provide a synergistic conductive boost due to their ability to exist in a multiple-oxidation state.
8. Metal aerogel alloys with inner transition metals/rare earth metals are a completely unexplored research area. Hybridizing *d*-block elements with *f*-block elements modifies the electronic structure of noble metal aerogels, affecting their selectivity towards the adsorption and chemisorption of intermediates, likely due to the *d-f* coupling.
9. Since its discovery in 2009, all research has focused on developing and comprehending the structure–property relationship predominantly with noble metal aerogels; there is very little literature on non-noble metal aerogels. This area is so crucial that using non-noble metal aerogels can lower the electrocatalyst cost by several orders. Therefore, more research on non-noble metal aerogels is suggested.
10. Surface modifications of metal aerogels, such as incorporating carbonaceous species, nitridation, sulfidation, phosphorylation, conducting polymers, and ILs could be one of the ways to enhance the activity and stability of metal aerogels, which would help the faster MOR kinetics and enhance electronic conductivity by modulating the electronic structure of metal aerogel.
11. In situ formation and growth of metal aerogels directly on the gas diffusion layers by either immersed chemical reduction methods or electrochemical reduction methods could be one of the effective strategies to transfer the metal aerogels directly on GDL, which could avoid further processing of the MA catalyst ink. This could be a valuable idea to pursue.

## Figures and Tables

**Table 1:** Acidic and alkaline DMFCs have their own advantages and disadvantages.

Acidic DMFCs	Alkaline DMFCs
Advantages	Advantages
Sluggish methanol oxidation reaction (MOR) kinetics Uses proton exchange membrane as solid electrolyte Admirable power density Fater H^+^ conduction Mature technology Requires noble metal catalysts for better anodic and cathodic reactions High catalyst cost	Relatively faster MOR kinetics Uses alkaline membrane Cathode is less sensitive to methanol Relatively lower CO poisoning issues Faster ORR kinetics at cathodes Noble metals are not necessary Low catalyst cost Less carbon and materials corrosions
Disadvantages	Disadvantages
Mixed potentials due to cathodic ORR and MOR High methanol fuel crossover Cathode catalyst poisoning Requires auxiliary metal to overcome CO poisoning of the Pt catalyst Requires noble metals for both anode and cathode electrocatalytic reactions High cost Caron supports corrosion	Slower ionic conduction Formation of CO_3_^2−^ when CO_2_ from air reacts with OH^−^ Low stability of alkaline membrane due to OH^−^ attack. Complicated H_2_O management system Less mature technology Lowe methanol crossover

**Table 2:** Kinetic and stability data of metal aerogel catalysts.

Metal Aerogel Catalysts	Reducing Agent/Gelation Time	BET/ECSA (m^2^ g^−1^)	Electrolyte	^a^ Mass Activity ^b^ Specific Activity	^a^ Mass Activity ^b^ Specific Activity vs. Standard Catalyst	Stability of the Catalyst	DMFC Power Density (mW cm^−2^)	Ref
Au-Pt aerogels	NaBH_4_/2–4 h	NA/53.9	1.0 M H_2_SO_4_ + 1.0 M CH_3_OH	^b^ 0.51 A mg^−1^	0.21 A mg^−1^	CA for 3600 s—3.8 times higher current retention than that of control samples	NR	[81]
Pt_3_Cu_1_ aerogel	NaBH_4_/8 h	38/116	0.5 M H_2_SO_4_ + 1 M CH_3_OH	^b^ 3.77A mg^−1^_Pt_	0.575 mA mg^−1^_Pt_	CA for 3600 s—8.8 times higher current retention than that of Pt/C catalyst	NR	[84]
2D Pt-Ni	NaBH_4_/12 h	NR/50.6	0.5 M H_2_SO_4_ + 0.5 M CH_3_OH	1.8 A mg^−1^	NR	NR	NR	[94]
NCDs-PtNi	NaBH_4_/12 h	29/99.4	0.1 M HClO_4_ + 1.0 M CH_3_OH	4.8 A mg^−1^_Pt_ 8.4 mA cm^−2^_Pt_	0.4 Amg^−1^_Pt_ 0.7 mA cm^−2^_Pt_ (Pt/C)	CV—5000 cycles—63.5% ECSA retention	NR	[98]
Pt_2_Bi_1_-10/1	NaBH_4_/NR	~21/NR	1.0 M KOH + 1.0 M CH_3_OH	3.05 A mg Pt^−1^	0.70 A mgPt^−1^ (Pt/C)	NR	NR	[93]
PtCu-HC	NaBH_4_/24 h	NR/26.9	0.5 M H_2_SO_4_ + 1 M CH_3_OH	1.30 A mg^−1^_Pt_ 4.83 mA cm^−2^	0.72 (Pt/C) and 0.61 (Pt-Ru/C) A mg^−1^_Pt_	Mass activity after the stability test 1.12 A mg^−1^_Pt_	NR	[145]
Au_1_-Pt_67_-Co_33_	NaBH_4_/12 h	47/35	0.5 M H_2_SO_4_ + 1 M CH_3_OH	1.033 A mg^−1^_Pt_	0.227 A mg^−1^_Pt_	CA for 2000 s—Maintained high current density	NR	[122]
Pt_1_Cu_1_Ir_0.04_	NaBH_4_/4 h @ 60 °C	24/66	1 M HClO_4_ + 1 M CH_3_OH	4.73 A mg^−1^_Pt_ 8.4 mA cm^−2^	1.12 A mg^−1^_Pt_ 3.24 mA cm^−2^	CV—100 cycles—80% retention ECSA	NR	[118]
HD-PtRuPd AA	NaBH_4_/6 h @ 45 °C	NR/33.9	0.5 M H_2_SO_4_ + 1 M CH_3_OH	2.42 A mg^−1^_Pt_	0.54 A mg^−1^_Pt_ (Pt/C)	CA—1800 s—high stability over Pt/C	60.4	[123]
Pt_3_Cu_3_Mo_0.5_ AA	NaBH_4_/12 h @ 20 °C	NR/35.5	0.5 M H_2_SO_4_ + 1 M CH_3_OH	1.7 A mg^−1^_Pt_ 4.7 mA cm^−2^	0.6 mA cm^−2^ (Pt/C)	CA—1800 s—high stability over Pt/C	12	[124]
PtPdCu MEAAs	NaBH_4_/NR	NR/45.9	0.1 M HClO_4_ + 1 M CH_3_OH	1.84 A mg^−1^_Pt+Pd_ 4.22 mA cm^−2^	0.565 mg^−1^_Pt_ 1.14 mA cm^−2^ (Pt/C)	CA—3600 s—high stability over Pt/C	35	[134]
PtBi_1.5_Co_0.2_Ni_0.2_Cu_0.2_ aerogel	NaBH_4_/NR	NR/NR	0.5 M H_2_SO_4_ + 1 M CH_3_OH	4.19 A mg^−1^_Pt_	0.33 A mg^−1^_Pt_ (Pt/C)	CA—3600 s—retention of 42.7% current	115	[131]
PtPdRuCuNi	NaBH_4_/6 h @ 20 °C	41.35/36.9	0.5 M H_2_SO_4_ + 1 M CH_3_OH	2.05 A mg^−1^_Pt_	0.55 A mg^−1^_Pt_ (Pt/C)	CA—3600 s—high stability over Pt/C	77.7	[132]
Bi decorated Pt aerogel	NaBH_4_/4 h	NR/125	1.0 M KOH + 1.0 M CH_3_OH	7.99 A mg^−1^_Pt_	0.99 A mg^−1^_Pt_	CA for 7200 s—1500 mA mg^−1^_Pt_	NR	[146]
PtRu aerogel	NaBH_4_/NR	47.6/NR	1.0 M KOH + 1.0 M CH_3_OH	6.531 A mg^−1^_Pt_	3.662 A mg^−1^_Pt_	CV—300 cycles—82.1%	NR	[147]
Au_50_Pt_50_ gel	NaBH_4_/24 h	58.7/NR	1.0 M KOH + 1.0 M CH_3_OH	11.57 A mg^−1^_Pt_	0.531 (Pt/C)	NR	NR	[144]
IL/Pd_3_Cu_1_	NaBH_4_/1 h	NR/116	1.0 M KOH + 1.0 M CH_3_OH	2.96 A mg^−1^_Pd_ 2.55 mA cm^−2^	8.5 and 3.2 times lower (Pd/C)	CA—3600 s—Maintained high current density	NR	[103]
Pd aerogel	Electrochemical Reduction	NR/102	1.0 M KOH + 1.0 M CH_3_OH	2.99 A mg^−1^_Pd_	0.64 A mg^−1^_Pd_ (Pd/C)	NR	NR	[106]
Pd_3_-(CoO_x_)1	Electrochemical Reduction	NR/58.72	1.0 M KOH + 1.0 M CH_3_OH	4.94 A mg^−1^_Pd_	10.5 times lower (Pd/C)	NR	NR	[108]
Pd_3_Sn MAs	N_2_H_4_/12 @ 60 °C	190/47.83	1.0 M KOH + 0.5 M CH_3_OH	0.67 A mg^−1^_Pd_ 0.56 mA cm^−2^	Lower than MAs (Pd/C)	CV—250 cycles—the ECSA reduced to 41.72 m^2^ g^Pd−1^	NR	[116]
Pt_4_Ru_1_Cu_5_ aerogels	NaBH_4_/2 h @ 60 °C	NR/56.5	1 M KOH + 1 M CH_3_OH	2.07 A mg_N_^−1^	0.74 A mg_N_^−1^	CA—3600 s—slowest current decay among these catalysts	NR	[117]
PtNi-2	NaBH_4_/12 h	28.4/71	1 M KOH + 1 M CH_3_OH	3.2 A mg^−1^_Pt_ 5.5 mA cm^−2^	NR	CV—100 cycles—78% retention ECSA	NR	[120]
Ag/Pt/Pd-2 aerogel	NaBH_4_/NR	140/NR	1 M KOH + 1 M CH_3_OH	3.178 A mg^−1^	4.1 (Pt/C) and 5.0 (Pd/C) times lower than MAs	CA—17 h—high stability over Pt/C and Pd/C	NR	[121]
Au/Ag/Pt aerogel	NaBH_4_/NR	125/NR	1 M KOH + 1 M CH_3_OH	1.962 A mg^−1^	2 times less than the MA catalyst (Pt/C)	CA—24 h—retention of 94% current	NR	[119]

NR = Not reported. ^a^ Mass Activity. ^b^ Specific Activity.

**Table 3:** MOR mass activity values of shape-controlled advanced catalysts, carbon-supported catalysts, and non-carbon-supported catalysts.

Catalyst	Electrolyte	Mass Activity (A mg^−1^)	Reference
Shape-controlled advanced catalysts			
PtCuNi nanohexapods	0.5 M H_2_SO_4_ + 1 M CH_3_OH	1.73	[148]
2D Pd-Fe-Pt Nanomeshes	0.1 M HClO_4_ + 0.5 M CH_3_OH	1.61	[149]
Au-PtNi DNPs	0.5 M H_2_SO_4_ + 0.5 M CH_3_OH	0.59	[150]
AuPtNi NSs	0.5 M H_2_SO_4_ + 1.0 M CH_3_OH	0.99	[151]
Pt-Au NWs	0.1 M HClO_4_ + 1.0 M CH_3_OH	1.04	[152]
PtCu branched nanocrystals	0.5 M H_2_SO_4_ + 1 M CH_3_OH	1.59	[153]
Au/Pt nanobowls	0.1 M H_2_SO_4_ + 1 M CH_3_OH	0.497	[154]
PtCuFe nanoframes	0.5 M H_2_SO_4_ + 2 M CH_3_OH	1.030	[155]
PtCo nanocrosses	0.5 M H_2_SO_4_ + 1 M CH_3_OH	0.692	[156]
PtCoRh SNWs	0.1 M HClO_4_ + 0.5 M CH_3_OH	1.480	[157]
P-PtNi CNC	0.5 M H_2_SO_4_ + 2 M CH_3_OH	0.380	[158]
Pt nanoribbons	1.0 M HClO_4_ + 1 M CH_3_OH	0.570	[159]
Pt_3_NiRh nanoflowers	1.0 M CH_3_OH + 0.5 M H_2_SO_4_	1.9	[160]
Pt_0.68_Cu_0.18_Ru_0.14_ NFs	0.5 M CH_3_OH + 0.1 M HClO_4_	0.82	[161]
Pt_3_Mn NWNs	0.1 M HClO_4_ + 0.5 M CH_3_OH	0.843	[162]
concave PtCo nanocrosses (PtCo CNCs)	0.1 M HClO_4_ + 0.5 M CH_3_OH	0.692	[156]
PtCuCo nanoframes	0.1 M HClO_4_ + 1 M CH_3_OH	1.66	[163]
Ni_0.20_Pt_0.80_ nanoflowers	0.5 M H_2_SO_4_ + 1 M CH_3_OH	2.2	[164]
Hierarchically skeletal Pt-Ni nanocrystals (HSNs)	0.5 M H_2_SO_4_ + 1 M CH_3_OH	2.17	[165]
Pt_3_Ni nanoflower	0.5 M H_2_SO_4_ + 1 M CH_3_OH	1.44	[160]
Ru1Pt*_n_*-SAA	1.0 M CH_3_OH + 1.0 M HClO_4_	2.805	[166]
U-PtNi NWs	0.1 M HClO_4_ + 0.1 M CH_3_OH	1.562	[167]
Pt_3_Fe NWs	0.1 M HClO_4_ + 0.5 M CH_3_OH	1.30	[168]
Sub-1 nm PtSn ultrathin sheet	0.2 M KOH + 0.2 M CH_3_OH	0.87	[169]
Pd_83_Ni_17_ Nanostructures	1.0 M KOH +1.0 M CH_3_OH	1.11	[170]
PdCu-5 nanocages	1.0 M KOH + 1.0 M CH_3_OH	1.09	[171]
Pd_3_Pb NSAs/C	1.0 M KOH + 1.0 M CH_3_OH	2.98	[172]
PdCo J-NWs	1.0 M KOH + 1.0 M CH_3_OH	1.205	[173]
o-PdH_0.43_@Pt/CP	1 M KOH + 1 M CH_3_OH	3.68	[174]
Pt flat structure	1.0 M KOH + 0.5 M CH_3_OH	1.53	[175]
Dendritic PtSnBi	1 M KOH + 0.5 M CH_3_OH	4.02	[175]
PtBi nanorings	1.0 M CH_3_OH + 1.0 M KOH	6.42	[176]
PtPdAg nanotrees	1.0 M CH_3_OH + 1.0 M KOH	1.69	[177]
Cu/Pd_7_Te_3_ NWs	1.0 M CH_3_OH + 1.0 M KOH	1.68	[178]
Carbon-supported MOR catalysts			
Au@PtCu/C	0.1 M HClO_4_ + 0.5 M CH_3_OH	0.93	[179]
Au-doped PtCu_2_/C	0.1 M HClO_4_ + 0.5 M CH_3_OH	2.30	[180]
PtCuAu/C	0.1 M HClO_4_ + 0.5 M CH_3_OH	0.85	[181]
PtCo NCs/C	0.5 M H_2_SO_4_ + 0.5 M CH_3_OH	1.20	[182]]
Pt–Fe–Mn CNC	0.5 M H_2_SO_4_ + 2 M CH_3_OH	0.172	[183]
Ru–Pt_3_Sn NCs	0.1 M HClO_4_ + 1 M CH_3_OH	0.740	[184]
PtNiNF-NGA	0.1 M HClO_4_ + 1 M CH_3_OH	1.647	[185]
Pt/SiC	0.5 M CH_3_OH + 0.5 M H_2_SO_4_	0.65	[186]
Pt NPs/Siloxene	1.0 M CH_3_OH + 0.5 M H_2_SO_4_	1.96	[187]
Pt NDs/Fe-N-S-rGO	0.5 M CH_3_OH + 0.5 M H_2_SO_4_	0.932	[188]
PtPdCr/C TNPs	0.1 M HClO_4_ + 0.5 M CH_3_OH	0.969	[189]
Pt_60_Mn_1.7_Co_38.3_/C	1.0 M CH_3_OH + 0.5 M H_2_SO_4_	0.67	[190]
Fe_21_Pt_66_Rh_13_/C	0.1 M HClO_4_ + 0.5 M CH_3_OH	3.90	[191]
Pt-MoO_3_/CNT-ALD	0.5 M H_2_SO_4_ + 1 M CH_3_OH	1.221	[192]
Pt/Ni(OH)_2_/NG	0.1 M HClO_4_ + 0.5 M CH_3_OH	2.99	[193]
P-Pt_0.24_Cu/C	0.1 M HClO_4_ + 0.5 M CH_3_OH	2.3	[194]
PtTeCu NSs/C	0.1 M HClO_4_ + 1.0 M CH_3_OH	4.9	[195]
Pt/Co@NCNTs-MC800-4	0.5 M H_2_SO_4_ + 0.5 M CH_3_OH	0.7	[196]
Cu@Pt/C	0.5 M H_2_SO_4_ + 0.5 M CH_3_OH	0.624	[197]
Pt/Ti_3_C_2_T_x_-rGO	0.5 M H_2_SO_4_ + 0.5 M CH_3_OH	0.776	[198]
Pt/C nanowires	0.5 M H_2_SO_4_ + 0.5 M CH_3_OH	0.112	[199]
PtPd/C nanowires	0.5 M H_2_SO_4_ + 0.5 M CH_3_OH	0.0662	[199]
Pd@Pt/C core-shell nanoparticles	0.5 M H_2_SO_4_ + 0.5 M CH_3_OH	0.185	[199]
Pd@Pt/C core-shell nanowires	0.5 M H_2_SO_4_ + 0.5 M CH_3_OH	0.212	[199]
Pd-NiO-Y/CNT	1.0 M NaOH + 1.0 M CH_3_OH	3.9	[200]
Pd_0.52_Ag/CNTs	0.5 M NaOH + 1.0 MCH_3_OH	0.72	[201]
Pt cluster/Ti_3_C_2_T_x_/C	1 M KOH + 1 M methanol	7.32	[202]
Single-atom Ni-Pt nanowires/C	1 M KOH + 1 M methanol	7.93	[203]
Pt_1_/RuO_2_/C	0.1 M KOH + 1 M methanol	6.77	[204]
PtPbBi nanosheets/C	1 M KOH + 1 M methanol	11.90	[205]
Au@PdPt nanorods/C	1 M KOH + 1 M methanol	4.83	[206]
Pt_5_Ce/C	1 M KOH + 1 M methanol	9.13	[207]
PtBi nanorings/C	1 M KOH + 1 M methanol	6.42	[176]
Pt@N,F-HCS	1 M KOH + 1 M methanol	2.28	[208]
Pd@CoNi/rG	1 M KOH + 1 M methanol	1.730	[209]
Co-N-C/Pt	1.0 M KOH + 3.0 M CH_3_OH	5.6	[210]
Pt_1_RuO_2_	0.1 M KOH + 1.0 M CH_3_OH	6.766	[204]
Non-carbon-supported MOR catalysts			
Pt–CuO–TiO_2_	0.5 M HClO_4_ + 1.0 M CH_3_OH	1.537	[211]
Pt/double-shelled C/TiO_2_	0.5 M H_2_SO_4_ + 1.0 M CH_3_OH	0.462	[212]
Pt-WO_3_@W/GNs	0.5 M H_2_SO_4_ + 1.0 M CH_3_OH	0.881	[213]
Pt/C-Au@CeO_2_-Pt	0.5 M H_2_SO_4_ + 1.0 M CH_3_OH	1.267	[214]
Pt-CeO_2_	0.5 M H_2_SO_4_ + 1.0 M CH_3_OH	2.04	[215]
PtP-Fe_2_O_3_@FeP/C-8h	0.5 M H_2_SO_4_ + 1.0 M CH_3_OH	0.831	[216]
Pt/MnO_2_-R	0.5 M H_2_SO_4_ + 2.0 M CH_3_OH	0.51	[217]
Pt/Ti_0.7_Cu0.3N	0.1 M HClO_4_ + 0.5 M CH_3_OH	0.84	[218]
Ti_0.75_Mo_0.25_N	0.1 M HClO_4_ + 0.5 M CH_3_OH	0.98	[219]
PtRu/TiO_2_-GA	0.5 M H_2_SO4 + 2.0 M CH_3_OH	0.564	[220]
PtP-Fe_2_O_3_@FeP/C-8h	1.0 M KOH + 1.0 M CH_3_OH	3.215	[216]
Pd_6_Ru_4_/TiO_2–1_	0.5 M NaOH + 1.0 M CH_3_OH	2.8	[221]
Ti/TiO_2_NTs/PAIn/Pd	1.0 M KOH + 1.0 M CH_3_OH	6.38	[222]
Pt-CoO@NPC@SnO_2_-1	0.1 M KOH + 1.0 M CH_3_OH	1.518	[223]
PtSnO_2_	1.0 M KOH + 1.0 M CH_3_OH	0.108	[224]
PdP/WO_3_	0.1 M KOH + 1.0 M CH_3_OH	0.734	[225]
Pt-Ce(CO_3_)OH/rGO	1.0 M KOH + 1.0 M CH_3_OH	0.540	[226]
Pt/CeO_2_	1.0 M KOH + 1.0 M CH_3_OH	0.904	[227]
Pt-CeO_2_	0.5 M KOH + 2.0 M CH_3_OH	1.45	[228]
Fe_2_O_3_	1.0 M KOH + 1.0 M CH_3_OH	3.737	[229]
Zr-MOF@PANI/Ni-NPs	1.0 M NaOH + 0.5 M CH_3_OH	0.291	[230]

**Table 4:** DMFC single-cell performance of the metal aerogel catalysts.

Anode Metal Aerogel Catalyst & Catalyst Loading	Cathode Catalyst & Catalyst Loading	Solid Electrolyte	Anode Anolyte	Electrode Area and Power Density	Ref.
IL/Pd_3_Cu_1_, 2 mg cm^−2^	20 wt.% Pt/C, 2 mg cm^−2^	Alkalline membrane	5 M KOH + 1 M CH_3_OH	5 cm^2^, 8 mW cm^−2^ at 25 °C	[103]
HD-PtRuPd, 0.65 mg_Pt_.cm^−2^	Pt/C (40 wt.%) 2 mg cm^−2^	Nafion 115	1 M CH_3_OH	1 cm^−2^, 60.4 mW·cm^−2^	[123]
Pt_3_Cu_3_Mo_0.5_, 0.85 mg_Pt_.cm^−2^	Pt/C (40 wt.%) 2 mg cm^−2^	Nafion 115	1 M CH_3_OH + 1 M C_2_H_5_OH	1 cm^−2^, 12.02 mW·cm^−2^	[124]
PtPdCu MEAAs 2 mg PtPd cm^−2^	Pt/C (40 wt.%) 2 mg cm^−2^	Nafion 115	0 M CH_3_OH	1 cm^−2^, 35 mW cm^−2^ at 65 °C	[134]
PtBi_1.5_Ni_0.2_Co_0.2_Cu_0.2_ HEAAs	Pt/C (40 wt.%) 2 mg cm^−2^	Nafion 115	1 M CH_3_OH	115 mW cm^−2^	[132]

## Data Availability

No new data were created or analyzed in this study. Data sharing is not applicable to this article.
